# Clinical efficacy and Safety of Baloxavir Marboxil compared with Oseltamivir against influenza virus in children: A systematic review and meta-analysis

**DOI:** 10.1371/journal.pone.0326777

**Published:** 2025-06-23

**Authors:** Ling Zhu, Li Zhong, Guidong Huang

**Affiliations:** 1 Department of Pharmacy, The First Affiliated Hospital of Guilin Medical University, Guilin, China; 2 School of Pharmacy, Clinical Pharmacy, Guilin Medical University, Guilin, China; 3 Phase I Clinical Drug Research Center, The First Affiliated Hospital of Guilin Medical University, Guilin, China; 4 Guang Xi Key Laboratory for Pharmaceutical Molecular Discovery and Druggability Optimization, School of Pharmacy, Guilin Medical University, Guilin, China; Shantou University Medical College, CHINA

## Abstract

**Objective:**

Comparing the clinical efficacy and safety of baloxavir marboxil and oseltamivir against influenza viruses in children, to provide theoretical references for clinical practice.

**Methods:**

A systematic search of PubMed, Embase, Web of Science, Cochrane Library, Epistemonikos, CNKI, Wipu.com, Wan Fang Database, and China Biomedical Literature Database for articles published up to December 25th, 2024, was conducted. After literature screening, data extraction, and quality evaluation, descriptive analysis was performed.

**Results:**

Eight papers were included, comprising three randomized controlled studies and Five cohort studies, involving 3141 patients (1745 in the baloxavir marboxil group and 1396 in the oseltamivir group). Meta-analysis revealed no significant differences in time to remission of influenza symptoms and duration of fever between the two groups. However, baloxavir marboxil demonstrated a significantly greater reduction in influenza virus titer and RNA load. Additionally, the incidence of adverse events was significantly lower with baloxavir marboxil (p = 0.03).

**Conclusions:**

Baloxavir marboxil appears more effective than oseltamivir in reducing viral load and is associated with fewer adverse events in children with influenza, while both drugs yield comparable effects in relieving symptoms. Given the limited number of included studies and absence of subgroup analyses, further well-designed trials are needed to corroborate these findings.

PROSPERO Registration Number: CRD42024565338

## 1. Introduction

Influenza seriously affects their health and quality of life, especially in young children and immunocompromised children, who are prone to develop severe cases after influenza due to the imperfect function of the immune system, these populations are also the most common to be resistant to neuraminidase inhibitors (NAIs) [1,2]. Flu vaccination is the most effective means of preventing the flu [3,4].In the United States, routine annual influenza vaccination is recommended for all persons 6 months of age and older who have no contraindications for the vaccine. In the United Kingdom, annual influenza vaccination is recommended for school-age children and children aged 2 to 3 years. However, vaccination is not fully effective and the rate of vaccination needs to be increased [5,6]. RCT evidence suggests that prompt antiviral treatment reduces the duration of influenza symptoms and fever in children, decreases influenza complications, and reduces influenza transmission. Therefore, early treatment with anti-influenza drugs is essential once children are infected with influenza.

Currently available antiviral drugs include amantadines, neuraminidase inhibitors (NAIs), and ribavirin (Liberin). Amantadine interferes with the early replication of the virus by preventing the virus from shedding its capsid and releasing nucleic acids and is no longer recommended for the treatment of influenza because current strains have shown significant resistance [7,8]. The most commonly used of the NAIs is oseltamivir (OS), which selectively binds to neuraminidase and prevents the spread of influenza virus by inhibiting the cleavage of salivary acid glycoproteins on the surface of host cells [9]. However, due to the persistence of drug-resistant strains in influenza viruses, The development of new antiviral drugs to treat influenza is urgently needed. Baloxavir Marboxil (BXM) is a novel single-dose oral capsule-dependent nucleic acid endonuclease inhibitor antiviral drug with a mechanism significantly different from that of conventional NAIs, which is hydrolyzed in the gastrointestinal tract, intestinal epithelial cells, blood, and liver by arylacetamide deacetylase to the antiviral activity of baloxavir acid. Baloxavir acid targets the endonuclease activity of the acidic protein subunit within the influenza virus RNA polymerase. Thereby acting as a key link in viral replication. The metabolite baloxavir acid inhibits the endonuclease activity of the acidic protein subunit of influenza virus RNA polymerase, thereby directly inhibiting viral replication and exerting anti-influenza viral effects [2,10]. Such a mechanism of action makes baloxavir marboxil a novel, highly effective, and targeted drug for influenza treatment. In February 2018, baloxavir marboxil was the first to be approved for marketing in Japan. In October of the same year, Baloxavir marboxil was approved by the Food and Drug Administration (FDA) in the U.S. for the treatment of influenza A and B. In April 2021, China also approved the marketing of baloxavir marboxil for use in patients 12 years of age and older who have been suffering from acute, uncomplicated influenza with influenza symptoms for less than 48 hours [11–13]. In March 2023, the use of baloxavir marboxil was further expanded when it was licensed for broader clinical use. Today, the drug is now available for the treatment of simple influenza A and B in children aged 5 years and older, bringing a whole new treatment option for pediatric influenza patients. This important advancement undoubtedly offers new hope for pediatric influenza patients and heralds more effective treatments. A previous study showed that [14], In early childhood influenza, the duration of fever was significantly shorter in the baloxavir marboxil than in the oseltamivir group. However, some studies show [15] that in pediatric patients aged 1 to 12 years with influenza, baloxavir marboxil and oseltamivir had a similar overall incidence of adverse reactions, similar duration of fever, and time to resolution of influenza symptoms, but baloxavir marboxil was more rapid in terms of decline in viral titer. A meta-analysis of three randomized controlled trials [16] indicated that, compared to oseltamivir, Baloxavir marboxil significantly reduced both the frequency of adverse events and the duration of influenza symptoms. The meta-analysis was constrained, though, since it only examined adult outpatients and included those whose clinical symptoms suggested they might have been infected with the influenza virus. Thus, for pediatric patients infected with influenza viruses, it is now unclear which treatment is better: oseltamivir or baloxavir marboxil.

Because of this, this study provides a comprehensive assessment of the clinical efficacy and safety of baloxavir marboxil and oseltamivir by systematically evaluating many studies to compare the clinical efficacy and safety of baloxavir marboxil with that of oseltamivir in pediatric patients with influenza viral infections, to facilitate the introduction of medications into the hospitals and the clinical decision-making of medications by providing an objective and trustworthy clinical basis for the introduction of hospital medications, and better guide the clinical practice of pediatric patients with influenza.

## 2 Materials and methods

### 2.1 Inclusion and exclusion criteria

#### 2.1.1 Research target.

(1) The included patients were diagnosed with influenza, including those presenting with clinical influenza-like symptoms or those diagnosed with influenza by laboratory testing; (2) Patients were ≤18 years of age; (3) No restrictions on gender, race or severity of illness

#### 2.1.2 Intervention.

The experimental group was given antiviral treatment with baloxavir marboxil and the control group was given antiviral treatment with oseltamivir.

#### 2.1.3 Outcome indicator.

The outcome indicators for this study include: (1) Clinical efficacy:①Time to remission of symptoms (TTAS) in patients with influenza was defined as the duration from the start of treatment to the assessed disappearance or remission of all influenza-related symptoms;②Time to regression of fever (TTRF) was defined as the time to return to a fever-free state (37.5°C);③ Change from baseline in 48-hour viral titer;④Change from baseline in viral RNA load at 48 hours (2) Security Indicators: Incidence of adverse events; incidence of serious adverse events.

#### 2.1.4 Type of study.

The main types of literature included were randomized controlled studies, retrospective observational studies, or cohort studies. Only articles published in Chinese or English were included.

#### 2.1.5 Exclusion criteria.

(1) Missing data from thesis (2) No available outcome indicators (3) Data duplication (4) Full text not available (5) Low quality of literature (6) Non-Chinese and English literature (7) Reviews, case reports, animal experiment type studies.

### 2.2 Literature search strategies

This systematic evaluation and meta-analysis strictly followed the guidelines of the Preferred Reporting Items for Systematic Reviews and Meta-Analyses (PRISMA) [17]. This research protocol has been registered with the international systematic prospective registration site PROSPERO, registration number CRD42024565338. In this study, systematic searches were conducted in PubMed, Embase, Web of Science, Cochrane, Epistemonikos, China Knowledge Network (CNKI), Wipo.com, Wan Fang Database, and China Biomedical Literature (CBL) databases by using a combination of free-word and subject-word searches. The search encompassed articles published from the inception of each database through December 25, 2024, gathering literature that satisfied the inclusion criteria. Search terms mainly include “influenza virus”; “children”; “baloxavir marboxil”; “baloxavir”; “Xofluza”; “BXM”; “S-033188”; “s-03344”; “Oseltamivir”; “Tamiflu”. The search strategies employed for all databases are comprehensively detailed in Supplementary Material Table S1.

### 2.3 Literature screening, data extraction, and literature quality assessment

EndNote 16.1 was used to independently screen the literature retrieved from different databases according to the inclusion and exclusion criteria. First, duplicates retrieved from different databases were eliminated by using the software’s automatic duplicate elimination function, Subsequently, the titles and abstracts of the remaining literature were reviewed individually based on the pre-established inclusion and exclusion criteria, and the literature that met the inclusion criteria was screened for full-text reading, and in the process of full-text reading, literature that was irrelevant to the topic of the study was eliminated according to the exclusion criteria once again. In case of disagreement, consensus was reached through a two-person discussion or consultation with a third researcher to ensure the accuracy and reliability of the findings. The data of the literature finally included in the analysis was organized with the help of Excel 2019. A standardized data extraction form was used to extract data from the included literature. Information about each literature was detailed, including the first author, publication time, study type, number of patients, patient age, baloxavir marboxil versus oseltamivir dosage, influenza virus type, and outcome metrics.

The Cochrane Risk of Bias Assessment Tool was utilized to assess the risk of bias in the randomized controlled trials that were included [18]. There were seven main areas: randomized sequence generation; allocation concealment; blinding of subjects and implementers; blinding of outcome assessment; incomplete outcome reporting; selective reporting; and other sources of bias. Three ratings of “low risk,” “high risk,” and “unclear” were then made [19]. The Newcastle-Ottawa scale was employed to evaluate the three components of quality assessment—selection, comparability, and outcomes—in retrospective observational studies for cross-sectional research. Except for intergroup comparability, the bias assessment was based on the semi-quantitative star system evaluation principle. Which could be rated up to two stars, and the rest of the entries were rated up to one star. A full score of nine stars was used, with higher scores suggesting a higher quality of the literature study. The judgment of bias was expressed as “unsatisfactory,” “satisfactory,” “good,” or “very good.” To be more precise, literature with a score between 0 and 3 is regarded as low quality, literature with a score between 4 and 6 as moderate quality, and literature with a score between 7 and 9 as excellent quality.

### 2.4 Grade evidence grading evaluation

The quality of evidence was assessed using GRADE profiler 3.6, with evidence classified into four levels: high, moderate, low, and very low. The assessment was based on five key factors: risk of bias, inconsistency, indirectness, imprecision, and publication bias.

### 2.5 Statistical analysis

A thorough meta-analysis was conducted using RevMan 5.4 software from the Cochrane Collaboration. During the evaluation process, the variables were subdivided into two categories, dichotomous and continuous, based on their nature to ensure the accuracy and reliability of the analysis. For dichotomous variables (in this study, the incidence of adverse events and the incidence of serious adverse events), the Odds ratio (OR) and its 95% confidence interval (CI) were used as effect analysis statistics; for continuous variables (in this study, the time to relief of influenza symptoms, duration of fever, change from baseline in the viral titer and viral RNA load on the second day), the mean was calculated. and change from baseline in viral RNA load) the mean difference (MD) and its 95% CI were calculated for continuous variables (in this study, time to resolution of influenza symptoms, duration of fever, change in viral titer from the next day, and change in viral RNA load from baseline) to assess the combined effect size. The heterogeneity level was assessed using the P-value and the I^2^ statistic. A P < 0.1 indicated heterogeneity among studies, while an I^2^ value of 50% served as the threshold for determining the significance of heterogeneity across studies. I^2^ ≥ 50% was considered to suggest the presence of heterogeneity, Heterogeneity was considered negligible from 0 to 40%, 30–60% indicated moderate heterogeneity, and 50 to 90% suggested greater heterogeneity. Therefore, the degree of inter-study heterogeneity can be effectively assessed and quantified by the P-value and I^2^ statistic [20]. A random effects model was applied if P < 0.1 or I^2^ > 50%; in the other case, a fixed effects model was applied.

## 3 Results

### 3.1 Literature screening results

**Fig 1:**
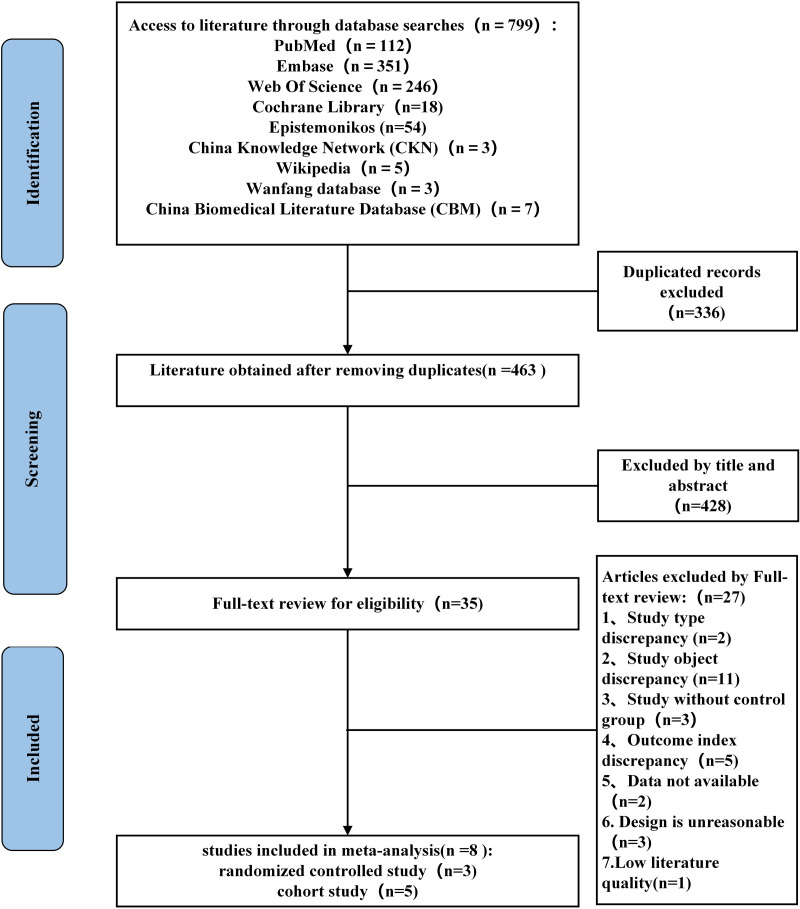
Flowchart of literature screening.

In the initial search, a total of 799 potentially relevant articles were identified. After removing 336 duplicate entries and screening the titles and abstracts of the remaining articles, 463 papers were subjected to further evaluation. Of these, 35 articles were selected for full-text review. Ultimately, eight papers [15,21–27] met the inclusion criteria after a thorough examination of their abstracts, titles, and full texts Fig 1 illustrates the detailed process and results of the literature screening.

### 3.2 Basic characteristics of literature

A total of Eight papers were included, including three randomized controlled trials [15,21,24], Cohort studies 5 [22,23,25–27], Published 2018–2024, The total sample size was 3141cases, including1745 cases in the baloxavir marboxil test group and 1396 cases in the oseltamivir control group. The basic characteristics of the study are shown in Table 1 and Table 2.

**Table 1:** Basic characteristics of the randomized controlled trials included in the Analysis.

Study	Country	Study duration	Study design	Number of eligible patients	Age (year)	Dosage	Outcome indicator	A subtype of influenza virus
Hayden, 2018 [21]	US Japan	December 2016 through March 2017	randomized controlled study	BXM : 456	Adults and Adolescents	BXM : 40mg OR 80 mg	①②⑤⑥	BXM : A (H1N1) n = 7, (H3N2)n = 393, B : n = 38; uncertain subtype n = 10
				OS : 377		OS : 75 mg, 2 times/d for 5 d		OS : A (H1N1)n = 2, A(H3N2)n = 332, B : n = 34; uncertain subtype n = 3
Baker, 2020 [15]	US Poland Spain Mexico Russia Costa Rica	January 11, 2017, to March 30, 2018	randomized controlled study	BXM:115	1-12	BXM: 40mg OR 80 mg	①②⑤⑥	BXM: A (H1N1)n = 18, A(H3N2)n = 48。 B : n = 5; uncertain subtype n = 4
				OS : 58		OS : 75 mg, 2 times/d for 5 d		OS : A (H1N1)n = 10, A(H3N2)n = 28 B, n = 2
Ison, 2020 [24]	Japan South Korea Philippines Taiwan UK Hungary Latvia Poland Romania Australia New Zealand, South Africa	During the 2018/2019 Northern Hemisphere Influence season	randomized controlled study	BXM : 388	12-18	BXM : 40mg : weight < 80 kg 80mg : weight ≥ 80 kg	①②③⑤⑥	BXM : A (H1N1)n = 28, A(H3N2)n = 182; B : n = 167; uncertain subtype n = 7
				OS : 389		OS : 75 mg, 2 times/d for 5 d		OS : A (H1N1)n = 35, A(H3N2)n = 190; B : n = 149; uncertain subtype n = 10

Note: ①: time to resolution of influenza symptoms; ②: duration of fever; ③: time to cessation of viral detoxification; ④: change in viral titer relative to baseline at 24 hours post-treatment; ⑤: change in viral titer relative to baseline at 48 hours post-treatment; ⑥: change in viral RNA load relative to baseline at 48 hours post-treatment.

**Table 2:** Basic characteristics of the cohort studies included in the analysis.

study	Country	Study duration	Study design	Number of eligible patients	Age (year)	Dosage	outcome indicator	A subtype of influenza virus
Saito, 2020 [25]	Japan	During the 2018/2019 Influence season	cohort study	BXM : 102	≤18	BXM : 10mg: ≤ 12 years old and weight 10 to 20 kg 20mg:≤ 12 years old and weight 20 to 40 kg 40mg:12–18 years or ≤ 12 years and ≥ 40 kg	①②	BXM : A (H1N1)n = 34, A (H3N2) n = 68
				OS : 52		OS : ≥37.5 Kg:75 mg,2 times/d for d <37.5 kg:2 mg/kg/d for 5 days		OS : A (H1N1)n = 17, A(H3N2)n = 35
Wagatsuma, 2022 [23]	Japan	During the 2019/2020 Influence season	cohort study	BXM : 100	≤18	BXM : 10mg: < 12 years old and weight 10 to 20 kg 20 mg:<12 years old and weight 20 to 40 kg 40mg: Patients aged 12–18 years or <12 years and weight ≥40 kg	①②	BXM : A (H1N1)n = 66, B : n = 34
				OS : 59		OS : 75 mg: ≥ 37.5 kg,2 times/day for 5 days2mg/kg/d: < 37.5 kg		OS : A (H1N1)n = 50, B : n = 9
Sato, 2021 [22]	Japan	During the 2018/2019 Influence season	cohort study	BXM : 20	<15	BXM : 10mg : Weight 10 to 20 kg 20mg : Weight 20 to 40 kg 40mg : Weight ≥ 40 kg	③⑤⑥	BXM : A, n = 20
				OS : 16		OS : Under 1 year : 2 mg/kg/dose, 2 times/day for 5 days Over 1 year: 3 mg/kg/dose, 2 times/day for 5 days		OS : A,n = 16,
Fujio, 2022 [26]	Japan	During the 2017/2018 2018/2019 2019/2020 Influence season	cohort study	BXM : 144	3-18	BXM : standard dose	②	BXM: A (H1N1)n = 60, A (H3N2) n = 42, B : n = 42
				OS : 91		OS : standard dose		OS: A (H1N1)n = 40 A (H3N2) n = 32 B : n = 19
Ge, X, 2024 [27]	China	March, 2023, to December, 2023	cohort study	BXM : 246	0-18	BXM : 40mg: Weight < 80 kg or<5 years 80mg: Weight ≥ 80 kg	②	BXM: A: n = 246
				OS : 246		OS : 75mg: ≥ 13 years, 2 times/day 30mg: < 13years, Weight≤15 kg, 2 times/day 45 mg: < 13years, Weight 15–23 kg, 2 times/day 60 mg: < 13years, Weight 23–40 kg, 2 times/day 75 mg: < 13years, Weight >40 kg, 2 times/day		OS: A: n = 246

Note: ①: time to resolution of influenza symptoms; ②: duration of fever; ③: time to cessation of viral detoxification; ④: change in viral titer relative to baseline at 24 hours post-treatment; ⑤: change in viral titer relative to baseline at 48 hours post-treatment; ⑥: change in viral RNA load relative to baseline at 48 hours post-treatment.

### 3.3 Literature quality assessment

**Fig 2:**
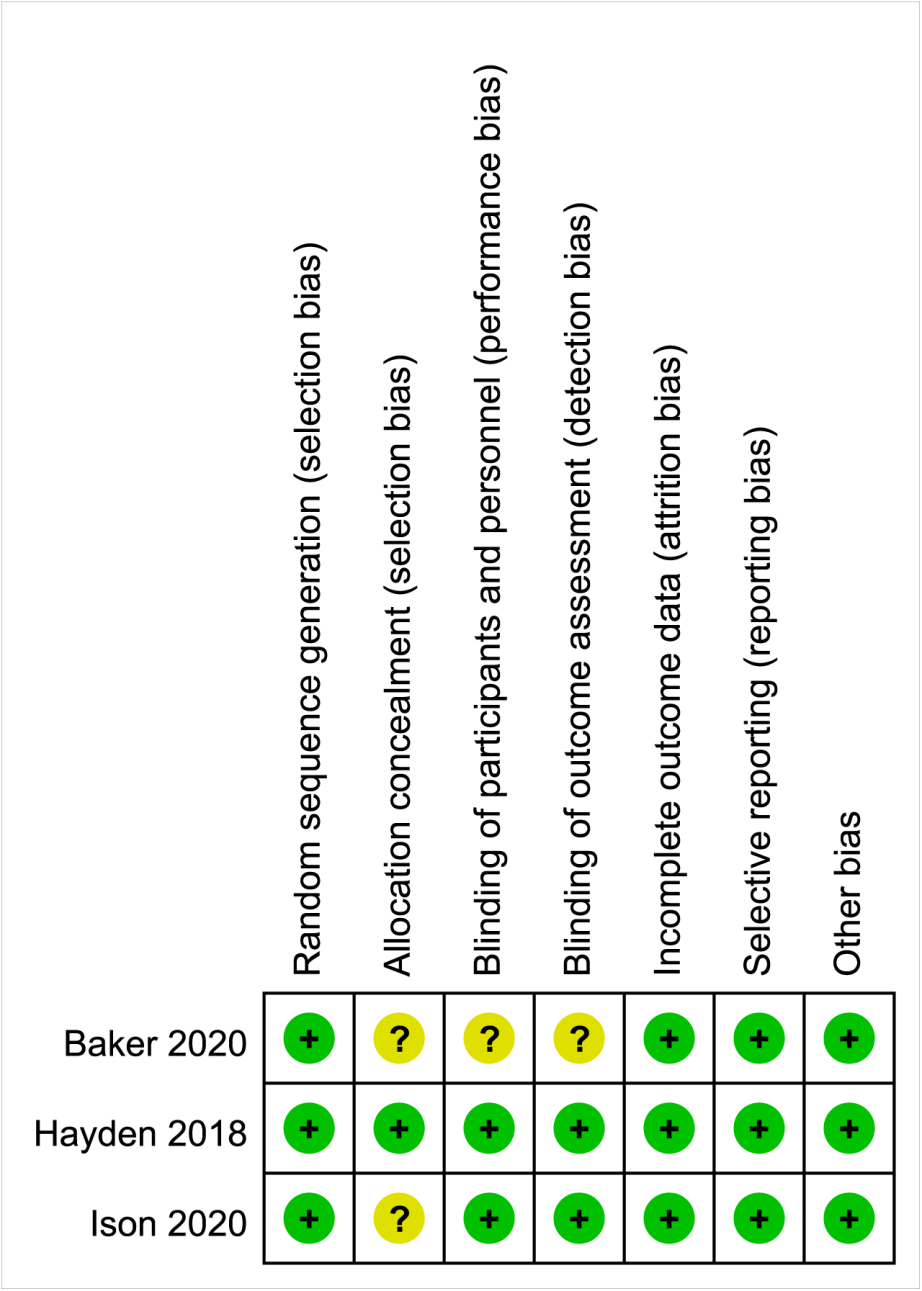
Diagram of risk of bias assessment for randomized controlled studies.

Of the three RCTs included, two [21,24] were grouped using a randomized numeric table method and were judged to be “low risk”. The remaining study [15] was a randomized block allocation method and was judged to be “low risk”; Both studies [15,24] did not specify whether allocation concealment was practiced and judged the risk to be “unclear”; One study [21], which did not specify whether subjects and investigators were blinded, was assessed as “unclear risk”, and the remaining studies were assessed as “low risk”; All included studies with complete and credible data on the evaluation indicators and no obvious selective reporting were judged to be “low risk”; the presence of other biases was not detected and was judged to be “low risk”. The risk of bias graph was plotted using Rev Man 5.4 software, and the specific risk of bias for each study is shown in Fig 2. Five cohort studies [22,23,25–27] The Newcastle-Ottawa Quality Assessment Scale (NOS) scores were “7,” “6,” “7,” “6,” and “7 “. For detailed scoring information, please refer to Supplementary Material Table S2.

### 3.4 Meta-analysis results

#### 3.4.1 Time to remission of flu symptoms.

**Fig 3:**
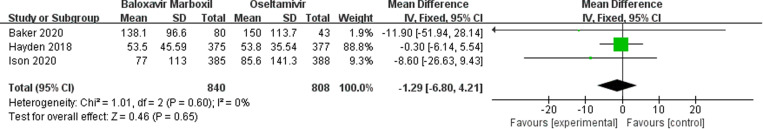
Forest plot of Meta-analysis of time to remission of influenza symptoms in randomized controlled studies.

**Fig 4:**
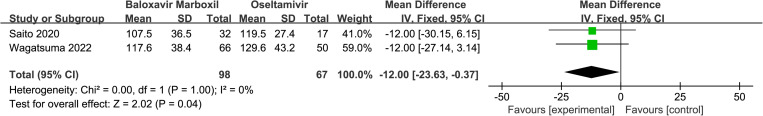
Forest plot of Meta-analysis of time to remission of influenza symptoms in cohort studies.

Three randomized controlled trials [15,21,24] all reported the time to remission of influenza symptoms. When compared with oseltamivir, these studies demonstrated no statistically significant heterogeneity in terms of symptom remission time (P = 0.60, I² = 0%). Using a fixed-effects model, the meta-analysis revealed that patients in the baloxavir marboxil group experienced a shorter time to remission of influenza symptoms compared to those in the oseltamivir group; however, this difference was not statistically significant [MD = −1.29, 95% CI (−6.80, 4.21), P = 0.65]. These findings are detailed in Fig 3. Two [23,25]out of the five cohort studies the time to remission of influenza symptoms, with no statistically significant heterogeneity observed between these studies when compared to oseltamivir (P = 1.00, I² = 0%). The meta-analysis revealed that patients treated with baloxavir marboxil experienced a significantly shorter time to symptom remission compared to those treated with oseltamivir [MD = −12.00, 95% CI (−23.63, −0.37), P = 0.04]. Detailed results are presented in Fig 4.

#### 3.4.2 Time to regression of fever.

**Fig 5:**
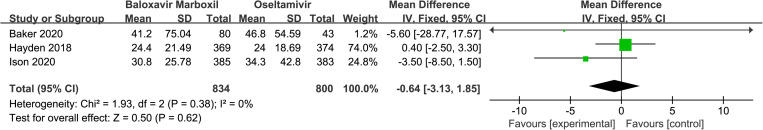
Forest plot of Meta-analysis of fever duration in randomized controlled studies.

**Fig 6:**
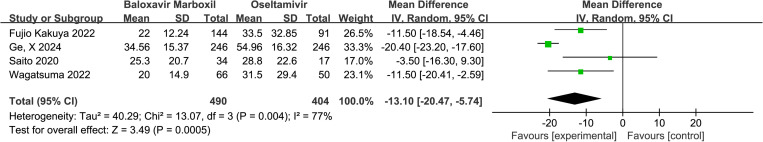
Forest plot of Meta-analysis of fever duration in cohort studies.

Three randomized controlled trials [15,21,24] reported on fever duration. Compared to oseltamivir, no statistically significant heterogeneity was observed across these studies (P = 0.38, I² = 0%). Using a fixed-effects model, the meta-analysis revealed that the duration of fever in patients treated with baloxavir marboxil was shorter compared to those treated with oseltamivir; however, this difference was not statistically significant [MD = –0.64, 95% CI (−3.13, 1.85), P = 0.62]. Detailed results are presented in Fig 5. Whereas four out of five cohort studies [23,25–27] reported fever duration, compared with oseltamivir, significant heterogeneity was observed among the studies (P = 0.004, I^2 ^= 77%), leading to the use of a random effects model for analysis. The meta-analysis results indicated that patients treated with baloxavir marboxil experienced a significantly shorter duration of fever compared to those treated with oseltamivir, with a statistically significant mean difference [MD = −13.10, 95% CI (−20.47, −5.74), P = 0.0005). Details are presented in Fig 6.

#### 3.4.3 Change in viral titer from baseline to the next day.

**Fig 7:**
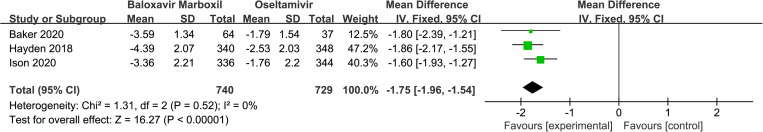
Forest plot of Meta-analysis of the change in viral titer from baseline to the next day in a randomized controlled study.

Three randomized controlled trials [15,21,24] all reported changes in viral titers from baseline to the next day in patients with influenza, with no statistically significant differences in heterogeneity between studies (P = 0.52, I^2^ = 0%). Using a fixed-effects model, Meta-analysis showed that the decrease in 48-hour viral titer from baseline was significantly higher in the baloxavir marboxil group than in the oseltamivir group, and the difference was statistically significant. [MD = –1.75,95%CI(−1.96, -1.54), P < 0.00001] Details are shown in Fig 7.

#### 3.4.4 Change in viral RNA load from baseline to the next day.

**Fig 8:**
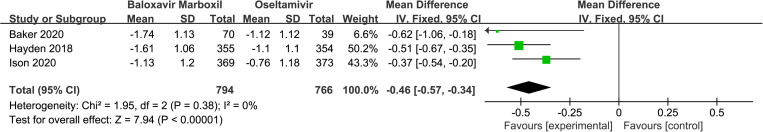
Forest plot of Meta-analysis of the change in viral RNA load from baseline to the next day in a randomized controlled study.

Changes in viral RNA load from baseline to the next day in patients with influenza were reported in three randomized controlled trials [15,21,24] with no statistically significant differences in heterogeneity between studies (P = 0.38, I^2^ = 0%). Using a fixed-effects model, Meta-analysis showed that the 48-hour viral RNA load decreased more significantly in the baloxavir marboxil group than in the oseltamivir group, and the difference was statistically significant[MD = –0.46, 95%CI(−0.57, -0.34), P < 0.00001] Details are shown in Fig 8.

#### 3.4.5 Incidence of any adverse reactions.

**Fig 9:**
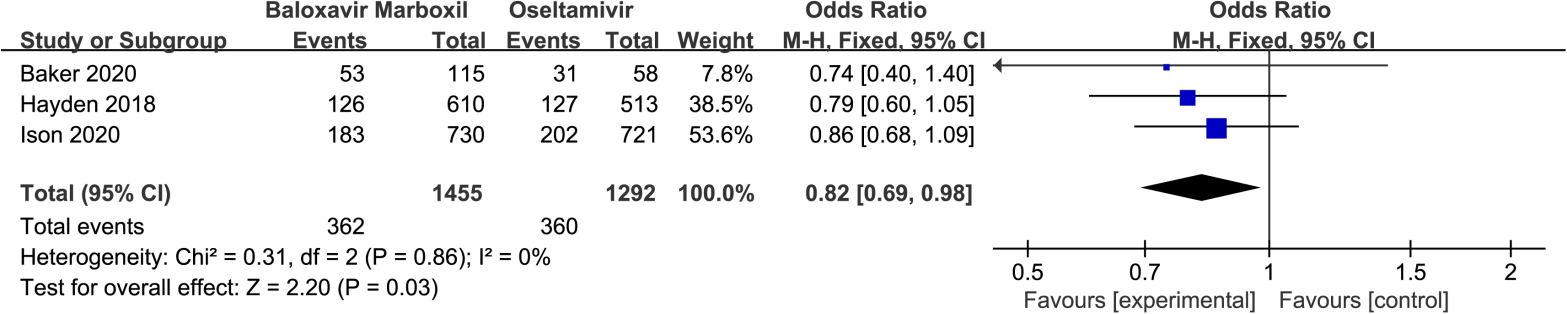
Forest plot of Meta-analysis of the incidence of any adverse reactions in randomized controlled studies.

Adverse events were reported in three randomized controlled studies [15,21,24]. 362 in the baloxavir marboxil trial group and 360 in the oseltamivir control group. The most common adverse reactions are characterized by nausea and vomiting, diarrhea, and bronchitis. These findings are summarized in Table 3. The incidence of adverse reactions was 24.88% (362/1455) in the baloxavir marboxil group and 27.87% (360/1292) in the oseltamivir group, with no statistically significant difference in heterogeneity between studies (P = 0.86, I^2^ = 0%). Using a fixed-effects model, Meta-analysis showed a statistically significant difference between the incidence of adverse reactions in patients in the baloxavir marboxil group and the oseltamivir group. [OR=0.82,95%CI(0.69,0.98), P = 0.03] Details results are presented in Fig 9.

**Table 3:** Adverse reaction statistics (cases).

	Hyden 2018		Baker 2020		Ison 2020	
	experimental group	control groups	experimental group	control groups	experimental group	control groups
Any	126	127	53	31	183	202
nausea and vomiting	13	22	7	9	20	34
diarrhea	18	11	6	1	20	23
bronchitis	16	18	3	1	21	30
tonsillitis	9	4	0	0	0	0
sinusitis	7	5	4	1	14	22
Elevated transaminases	6	7	0	0	0	0
dizziness and headaches	8	5	0	0	0	0
leucopenia	0	1	0	0	0	0
otitis media	0	0	3	4	0	0
cough	0	0	3	1	0	0
earache	0	0	1	2	0	0
upper respiratory infection	0	0	5	2	0	0
Serious adverse event	2	0	0	0	5	8

#### 3.4.6 Incidence of serious adverse reactions.

**Fig 10:**
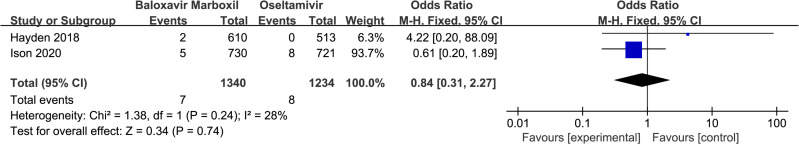
Forest plot of Meta-analysis of incidence of serious adverse reactions in randomized controlled studies.

Two of [15,24] the three randomized controlled studies reported serious adverse events, reported serious adverse events, seven in the baloxavir marboxil group, and eight in the oseltamivir control group. The incidence of serious adverse reactions was 0.52% (7/1340) in the baloxavir marboxil group and 0.65% (8/1234) in the oseltamivir group, with no statistically significant difference in heterogeneity between studies (P = 0.24, I^2^ = 28%). Using a fixed-effects model, Meta-analysis showed that the difference in the incidence of serious adverse reactions between patients in the baloxavir marboxil group and the oseltamivir group was not statistically significant [OR=0.84,95%CI(0.31,2.27), P = 0.74] Details are shown in Fig 10.

#### 3.4.7 GRADE evidence classification.

The evidence levels for the outcome measures included in the meta-analysis were assessed, with the studies categorized as randomized controlled trials (RCTs) and cohort studies. For RCTs, the evidence was initially classified at the highest level, with any downgrading determined according to the five GRADE criteria. The results showed that, in RCTs, the quality of evidence for time to relief of flu symptoms, duration of fever, incidence of any adverse events, and incidence of serious adverse events was moderate, while the evidence for viral titer and viral RNA load was high. In cohort studies, the quality of evidence for both the time to relief of flu symptoms and the duration of fever was rated as very low. The detailed assessments are provided in Supplementary Materials Table S2.

## 4 Discussion

Children have a high prevalence of influenza, with an annual incidence rate of about 30 percent of the total number of influenza patients, and the risk of severe influenza is high, and untimely treatment is prone to develop into severe cases [28,29]. Anti-influenza drugs are important for controlling influenza in children, and the current guidelines recommend oseltamivir as the commonly used drug, and baloxavir marboxil as a new drug that increases the choice of medication for influenza in children [30,31]. To further expand the application of baloxavir marboxil, this study provides a more comprehensive reference for clinical use by comparing and analyzing the clinical efficacy and adverse reactions of baloxavir marboxil and oseltamivir in treating influenza in children. Three randomized controlled studies and five cohort studies involving a total of 3,141 pediatric influenza patients were included in this study. This study has the following findings: (1) Clinical efficacy of baloxavir marboxil is comparable to oseltamivir; (2) Antiviral activity of baloxavir marboxil is superior to oseltamivir; (3) baloxavir marboxil has a better safety profile compared to oseltamivir.

In randomized controlled studies, baloxavir marboxil improved influenza symptoms and fever duration in children faster than oseltamivir, but there were no statistical differences. This finding was consistently demonstrated in the included studies. These findings are consistent with the results of previous Bayesian network meta-analyses by researchers such as Taieb [32] which suggests that baloxavir marboxil is as effective as oseltamivir, palamivir, and zanamivir. Nevertheless, only one phase II and phase III randomized controlled trial investigating the efficacy and safety of baloxavir marboxil as an antiviral agent for the treatment of healthy adult patients with influenza was included in this network meta-analysis. In contrast, three recently published randomized controlled studies in children were included in this study, thus contributing to further confirmation of the clinical efficacy of baloxavir marboxil against influenza virus in children. In the cohort study, baloxavir marboxil improved influenza symptoms and fever duration in children faster than oseltamivir and was statistically different. This may stem from the fact that cohort studies and randomized controlled studies differ essentially in experimental design and implementation, leading them to reach different conclusions. Cohort studies typically look at the relationship between exposure and outcome over a longer period, whereas randomized controlled studies assess the effect of interventions through the principle of allocation, and these differences can lead to inconsistent findings.

In terms of reducing viral levels on the second day of dosing, the baloxavir marboxil group was statistically more effective than the oseltamivir group in reducing both viral RNA load and viral titer. This result is in agreement with previous studies by Shiraishi [10] et al researchers and Taieb [32] et al researchers. This may stem from the different mechanisms of action of baloxavir marboxil and oseltamivir, with baloxavir marboxil acting on the process of viral replication and oseltamivir on the process of viral release. Baloxavir marboxil is a precursor drug that is hydrolyzed in the body to the active form of baloxavir. Baloxavir inhibits influenza virus mRNA synthesis by inhibiting the endonuclease activity of the PA subunit of influenza virus RNA polymerase, thus blocking the proliferation of influenza virus. Briefly, the mechanism of action of baloxavir marboxil is to directly reduce viral replication, whereas oseltamivir has a slightly different mechanism of action, which is to inhibit the neuraminidase activity of influenza A and B viruses, thereby reducing viral transmission. Thus, while both baloxavir marboxil and oseltamivir are effective antiviral agents, baloxavir marboxil may exhibit greater antiviral activity by directly reducing viral replication.

In terms of clinical safety, only three of the eight included studies [15,21,24] reported adverse reactions. Studies by Hyden et al [21] and Baker et al [15]showed that the most common adverse reactions were gastrointestinal reactions in the form of diarrhea, nausea, and vomiting, most of which occurred on day 1 or day 2 of treatment and resolved spontaneously. A study by Ison et al [24] showed that the most common adverse effects were sinusitis, bronchitis, nausea, and vomiting with diarrhea. The results of this study showed a statistically significant difference in the incidence of adverse reactions between the baloxavir marboxil group and the oseltamivir group, suggesting that baloxavir marboxil has a better safety profile compared to oseltamivir. Consistent with the results of a previous meta-analysis study by Liu, J. W et al investigators [33]. This study demonstrated that baloxavir marboxil had the lowest risk of adverse effects compared to oseltamivir, zanamivir, and palamivir. In terms of serious adverse reactions, only two studies [15,24] in the included literature reported serious adverse reactions, and none of these serious adverse reactions resulted in death. In one of the studies by Ison [24] 5 out of 730 patients treated with baloxavir marboxil experienced serious adverse reactions, but all of them were indicated by specific symptoms. 8 serious adverse reactions occurred in 721 patients using oseltamivir; two pediatric patients presented with elevated transaminases, both of which were thought to be related to the therapeutic agent, and the remaining serious adverse reactions were thought to be due to other causes. In a study by Hayden [21], 2 of 610 patients treated with baloxavir marboxil experienced serious adverse reactions, but no serious adverse reactions were observed in patients treated with oseltamivir. The results of this study showed that there was no significant difference between baloxavir marboxil and oseltamivir in terms of serious adverse effects; therefore, overall, baloxavir marboxil is considered to have a better safety profile in the treatment of influenza in children compared to oseltamivir. This aligns with the previous meta-analysis by Kuo YC [16].

Based on the available data, this study demonstrates that baloxavir marboxil exhibits good clinical efficacy and safety in the treatment of pediatric influenza, providing a solid foundation for its further clinical application. Currently approved indications demonstrate that oseltamivir is appropriate for children aged ≥1 year. Baloxavir marboxil has been approved by the FDA in the United States and China for children aged ≥5 years, whereas in the European Union and Japan, it is approved for children aged ≥1 year. However, in China, the use of baloxavir marboxil in children under 5 years of age is considered off-label due to limited safety and efficacy data, and thus it is generally not recommended for this population. For children under 5 years with mild influenza, oseltamivir is typically recommended as the first-line treatment. Based on the 2024 influenza surveillance data in China, there has been an observed increase in resistance to oseltamivir among influenza strains, while baloxavir marboxil continues to demonstrate high sensitivity. In clinical settings, baloxavir marboxil can be considered a first-line therapeutic option for pediatric patients with both mild and severe influenza, particularly when administered within 24 to 48 hours of symptom onset. Baloxavir marboxil has been shown to significantly reduce the duration of illness and alleviate symptoms more effectively than oseltamivir, exhibiting superior efficacy in decreasing viral load and viral RNA levels. For children with severe influenza, baloxavir marboxil may be incorporated into a comprehensive treatment strategy but often necessitates additional interventions such as hospitalization, respiratory support, or shock management.

This meta-analysis systematically evaluated the association between baloxavir marboxil and oseltamivir in the treatment of influenza in children, yielding significant conclusions. The findings demonstrated that baloxavir marboxil exhibits superior safety and antiviral activity compared to oseltamivir, providing strong support for its clinical use in pediatric influenza patients. To the best of our knowledge, this is the first comprehensive meta-analysis addressing the clinical efficacy and safety risks of baloxavir marboxil versus oseltamivir in this population. Despite the relatively systematic literature search and analysis, this study has several limitations: First, the limited number of included studies. The meta-analysis incorporated a small number of studies, several of which had modest sample sizes. This restriction may have reduced the statistical precision of the pooled results, resulting in wide confidence intervals that impact the accuracy and reliability of the findings. Second, the lack of subgroup analysis. Due to insufficient detailed data, we were unable to conduct subgroup analyses based on age groups, influenza virus subtypes, or comorbid conditions. This limitation might affect the generalizability of the conclusions to specific populations. Third, potential publication bias. The included studies provided limited discussion of potential publication bias, which may have resulted in an overestimation or underestimation of the observed effects. Finally, there is insufficient long-term data regarding baloxavir marboxil. This antiviral was introduced to the Chinese market recently, and a paucity of studies exists concerning its resistance patterns, reinfection rates, transmissibility, and long-term adverse effects. The lack of comprehensive data restricts the ability to thoroughly evaluate its long-term safety profile.

To address these limitations and further validate the clinical utility of baloxavir marboxil, future studies should focus on the following aspects: 1. Larger sample sizes and broader study scope: Conduct large-scale, multicenter randomized controlled trials (RCTs) to enhance the reliability and generalizability of the findings. 2. Subgroup Analyses: Investigate potential variations in efficacy and safety across different age groups, influenza virus subtypes, and children with preexisting conditions to offer more precise and targeted recommendations for clinical practice in specific patient populations. 3. Long-term outcomes research: Assess the development of resistance, evaluate reinfection risks, and investigate long-term adverse events associated with baloxavir marboxil to establish a comprehensive safety profile for its extended use. 4. Special Population Studies: Conduct rigorous and high-quality research in children under 5 years of age to evaluate the safety and efficacy of baloxavir marboxil in this population, particularly in cases of off-label use. This will provide robust scientific evidence to support its clinical application and inform guidelines for pediatric use.

## 5 Conclusions

This systematic review and meta-analysis indicates that baloxavir marboxil is associated with greater efficacy in reducing viral load and demonstrates a more favorable safety profile than oseltamivir in pediatric influenza treatment. Both agents show comparable effectiveness in symptom relief, yet the overall evidence supports the clinical potential of baloxavir marboxil as a promising alternative antiviral.

However, these conclusions should be interpreted in light of several limitations, including the small number of included studies, insufficient subgroup data, and lack of long-term safety outcomes. Future high-quality randomized controlled trials with adequate sample sizes, stratified analyses, and extended follow-up are essential to validate these findings and inform clinical guidelines.

## Supporting information

S1 Table Search strategies used for each electronic database, and list of excluded studies with reasons. (DOCX)

S2 Table Supplementary materials, including Newcastle-Ottawa Scale scores, RCT quality assessments, and GRADE summary for clinical outcomes. (DOCX)

S3 Table Outcome data analyzed from included randomized controlled trials and cohort studies, including comparisons of time to symptom remission, fever duration, viral load, and adverse event incidence. (DOCX)

S4 File PRISMA 2020 checklist for reporting items in the systematic review and meta-analysis. (PDF)
