# Supplementary material for: Clinical efficacy and Safety of Baloxavir Marboxil compared with Oseltamivir against influenza virus in children: A systematic review and meta-analysis
Source: PLoS One. 2025 Jun 23;20(6):e0326777. doi: 10.1371/journal.pone.0326777 (PMC12185026; doi:10.1371/journal.pone.0326777)
Supplement: S1 Table — (DOCX) [file pone.0326777.s001.docx]

**Supplementary** **Material** **Table S1**

**The search strategy of database search**

| **PubMed** | | |
| --- | --- | --- |
| ID | Search Terms | Results |
| #1 | "Orthomyxoviridae"[Mesh] | 66020 |
| #2 | ((((((Orthomyxoviridae[Title/Abstract]) OR (Influenza Viruses[Title/Abstract])) OR (Influenza Virus[Title/Abstract])) OR (Orthomyxoviruses[Title/Abstract])) OR (Orthomyxovirus[Title/Abstract])) OR (Myxoviruses[Title/Abstract])) OR (Myxovirus[Title/Abstract]) | 47020 |
| #3 | ("Orthomyxoviridae"[Mesh]) OR (((((((Orthomyxoviridae[Title/Abstract]) OR (Influenza Viruses[Title/Abstract])) OR (Influenza Virus[Title/Abstract])) OR (Orthomyxoviruses[Title/Abstract])) OR (Orthomyxovirus[Title/Abstract])) OR (Myxoviruses[Title/Abstract])) OR (Myxovirus[Title/Abstract])) | 79966 |
| #4 | "Oseltamivir"[Mesh] | 3380 |
| #5 | (oseltamivir[Title/Abstract]) OR (Tamiflu[Title/Abstract]) | 4738 |
| #6 | ("Oseltamivir"[Mesh]) OR ((oseltamivir[Title/Abstract]) OR (Tamiflu[Title/Abstract])) | 5460 |
| #7 | "baloxavir" [Supplementary Concept] | 228 |
| #8 | (baloxavir[Title/Abstract]) OR (xofluza[Title/Abstract]) | 383 |
| #9 | ("baloxavir" [Supplementary Concept]) OR ((baloxavir[Title/Abstract]) OR (xofluza[Title/Abstract])) | 391 |
| #10 | ((("Orthomyxoviridae"[Mesh]) OR (((((((Orthomyxoviridae[Title/Abstract]) OR (Influenza Viruses[Title/Abstract])) OR (Influenza Virus[Title/Abstract])) OR (Orthomyxoviruses[Title/Abstract])) OR (Orthomyxovirus[Title/Abstract])) OR (Myxoviruses[Title/Abstract])) OR (Myxovirus[Title/Abstract]))) AND (("Oseltamivir"[Mesh]) OR ((oseltamivir[Title/Abstract]) OR (Tamiflu[Title/Abstract])))) AND (("baloxavir" [Supplementary Concept]) OR ((baloxavir[Title/Abstract]) OR (xofluza[Title/Abstract]))) | 112 |
| **Web Of Science** | | |
| ID | Search Terms | Results |
| #1 | s-03344 (Topic) OR s-033188 (Topic) OR BXM (Topic) OR xofluza (Topic) OR baloxavir (Topic) OR baloxavir marboxil (Topic) and Preprint Citation Index (Exclude – Database) | 718 |
| #2 | influenza virus (Topic) OR human influenza virus (Topic) OR influenza viruses (Topic) OR influenzavirus a, b (Topic) OR myxovirus influenza (Topic) OR virus, influenza (Topic) and Preprint Citation Index (Exclude – Database) | 198280 |
| #3 | oseltamivir (Topic) OR tamiflu (Topic) OR tamivil (Topic) OR segosana (Topic) OR ro640796 (Topic) OR kewei (drug) (Topic) and Preprint Citation Index (Exclude – Database) | 10448 |
| #4 | #1 AND #2 AND #3 and Preprint Citation Index (Exclude – Database) | 246 |
| **Embase** | | |
| ID | Search Terms | Results |
| #1 | 'baloxavir marboxil'/exp | 484 |
| #2 | 'baloxavir marboxil':ab,ti OR rg6152:ab,ti OR 's 033188':ab,ti OR 's 33188':ab,ti OR s033188:ab,ti OR s33188:ab,ti OR xofluza:ab,ti OR baloxavir:ab,ti | 436 |
| #3 | #1 OR #2 | 671 |
| #4 | 'oseltamivir'/exp | 15418 |
| #5 | oseltamivir:ab,ti OR ebilfumin:ab,ti OR 'en 241104':ab,ti OR en241104:ab,ti OR enzamir:ab,ti OR fluvir:ab,ti OR 'gs 4104':ab,ti OR 'gs 4104 002':ab,ti OR 'hgp 0919':ab,ti OR 'hip 1403':ab,ti OR (kewei:ab,ti AND drug:ab,ti) OR 'oseltamivir phosphate':ab,ti OR 'ro 64 0796':ab,ti OR segosana:ab,ti OR tamiflu:ab,ti OR tamivil:ab,ti | 6654 |
| #6 | #4 OR #5 | 16109 |
| #7 | 'influenza'/exp | 123161 |
| #8 | 'infection caused by influenza virus':ab,ti OR 'influenza':ab,ti OR 'influenza infection':ab,ti OR 'influenza syndrome':ab,ti OR 'influenza virus infection':ab,ti OR 'influenza, human':ab,ti OR 'influenzavirus a, b':ab,ti OR 'myxovirus influenza':ab,ti OR 'influenza virus':ab,ti | 171049 |
| #9 | #7 OR #8 | 201408 |
| #10 | #3 AND #6 AND #9 | 351 |
| **Cochrane Library** | | |
| ID | Search Terms | Results |
| #1 | MeSH descriptor: [Orthomyxoviridae] explode all trees | 1391 |
| #2 | (Influenza Virus):ti,ab,kw OR (Myxoviruses):ti,ab,kw OR (Influenza Viruses):ti,ab,kw OR (Orthomyxovirus):ti,ab,kw OR (Orthomyxoviruses):ti,ab,kw | 3867 |
| #3 | #1 or #2 | 3933 |
| #4 | MeSH descriptor: [Oseltamivir] explode all trees | 317 |
| #5 | (oseltamivir):ti,ab,kw OR (Tamiflu):ti,ab,kw OR (GS4071):ti,ab,kw OR (GS4104):ti,ab,kw | 671 |
| #6 | #4 or #5 | 671 |
| #7 | (baloxavir marboxil):ti,ab,kw OR (xofluza):ti,ab,kw OR (s33188):ti,ab,kw OR (s033188):ti,ab,kw OR (rg6152):ti,ab,kw | 50 |
| #8 | #3 and #6 and #7 | 18 |
| **Epistemonikos** | | |
| ID | Search Terms | Results |
| #1 | (title:(influenza virus) OR abstract:(influenza virus)) | 34808 |
| #2 | (title:(baloxavir) OR abstract:(baloxavir)) | 200 |
| #3 | (title:(oseltamivir) OR abstract:(oseltamivir)) | 3414 |
| #4 | #1 AND #2 AND #3 | 54 |
| **SinoMed**(**CBM)** | | |
| ID | Search Terms | Results |
| #1 | "Influenza, human" | 14977 |
| #2 | "Influenza" [Common Field: Intelligent] OR "Influenza Epidemic" [Common Field: Intelligent] OR "Human Influenza" [Common Field: Intelligent] OR "Human Influenza" [Common Field: Intelligent] | 47441 |
| #3 | #1 OR #3 | 47441 |
| #4 | ("Oseltamivir" [unweighted: expanded]) AND "Oseltamivir" [unweighted: expanded] | 1134 |
| #5 | "Oseltamivir" [Common Field: Intelligent] OR "GS4071" [Common Field: Intelligent] OR "Tamiflu" [Common Field: Intelligent] OR "GS" [Common Field: Intelligent] AND "4104" [Common Field: Intelligent] OR "GS4104" [Common Field: Intelligent] AND "GS-4104" [Common Field: Intelligent] | 1995 |
| #6 | #4 OR #5 | 1995 |
| #7 | "Baloxavir marboxil" [Common Field: Intelligent] OR "Sufuda" [Common Field: Intelligent] OR "s-033188" [Common Field: Intelligent] OR "s-03344" [Common Field: Intelligent] | 19 |
| #8 | #3 AND #5 AND #6 | 7 |
| **CNKI** | | |
| ID | Search Terms | Results |
| #1 | (Subject: Influenza) OR (Subject: Epidemic Influenza) | 83371 |
| #2 | (Subject: Baloxavir marboxil) OR (Subject: Sufuda) OR (Subject: s-033188) OR (Subject: s-03344) | 23 |
| #3 | (Subject: Oseltamivir) OR (Subject: Tamiflu) OR (Subject: GS4071) OR (Subject: Tamiflu) OR (Subject: GS4104) | 2920 |
| #4 | #1 AND #2 AND #3 | 3 |
| **WanFang** | | |
| ID | Search Terms | Results |
| #1 | Subject: (Influenza) or All: (Influenza) | 101150 |
| #2 | All: (Mabaloxavir) or All: (Sufudah) or All: (s-033188) or All: (s-03344) | 33 |
| #3 | All: (Oseltamivir) or All: (Tamiflu) or All: (GS4071) or All: (GS4104) | 4697 |
| #4 | #1 AND #2 AND #3 | 3 |
| **WeiPu** | | |
| ID | Search Terms | Results |
| #1 | (Title or Keywords = Influenza OR Title or Keywords = Influenza A) | 61009 |
| #2 | (Title or Keywords = Baloxavir marboxil OR Title or Keywords = Sufuda) | 19 |
| #3 | (Title or Keywords = Oseltamivir OR Title or Keywords = Tamiflu) | 1712 |
| #4 | #1 AND #2 AND #3 | 5 |

**List of Excluded Studies with Reasons**

|  | **Citation** | **Reason** |
| --- | --- | --- |
| [1] | Zhang C, Yuan M, Rong W, Du H, Li X, Ji T, et al. Synergistic effects of Lianhuaqingwen in combination with Oseltamivir and Baloxavir against seasonal influenza virus: In vitro and in vivo assessment [J]. Journal of ethnopharmacology, 2025, 338(Pt 2): 119091. | Wrong population |
| [2] | Wan Qian, Ren Jinmei, Shen Yi. "You Should Know" about the Selection of Anti-Influenza Virus Drugs [J]. 2024, J China Food and Drug Administration. | wrong type of research |
| [3] | Jiang Tingting, Zhang Ni, Su Hui, Li Yanping, Liu Yao. Mining and Analysis of Adverse Event Signals of Oseltamivir, Zanamivir and Baloxavir Marboxil [J]. 2024, 35J China Pharmacy. | Wrong population |
| [4] | He Lin, Chen Jia, Wang Shuangmei, Bian Yuan. A safety study of anti-influenza virus drugs baloxavir marboxil and oseltamivir in the real world [J]. 2024, 44 Chinese Journal of Hospital Pharmacy. | wrong type of research |
| [5] | Zhou Y, Jin L, Lai X, Li Y, Sheng L, Xie G, et al. Adverse events related to oseltamivir and baloxavir marboxil in the treatment of influenza virus: A pharmacovigilance study using the FAERS database [J]. PloS one, 2024, 19(11): e0308998. | wrong type of research |
| [6] | Wei W, Huang L, Bai Y, Chang E, Liu J. The real-world safety of oseltamivir and baloxavir marboxil in children: a disproportionality analysis of the FDA adverse event reporting system [J]. Frontiers in pharmacology, 2024, 15. | wrong type of research |
| [7] | Taniguchi K, Noshi T, Omoto S, Sato A, Shishido T, Matsuno K, et al. The impact of PA/I38 substitutions and PA polymorphisms on the susceptibility of zoonotic influenza A viruses to baloxavir [J]. Archives of virology, 2024, 169(2): 29. | Wrong population |
| [8] | Takazono T, Ito G, Hosogaya N, Iwanaga N, Komeda T, Kobayashi M, et al. Comparison of the Effectiveness of Baloxavir and Oseltamivir in Outpatients With Influenza B [J]. Influenza and other respiratory viruses, 2024, 18(9): e70002. | Wrong population |
| [9] | Sun Y, Wagatsuma K, Saito R, Sato I, Kawashima T, Saito T, et al. Duration of fever in children infected with influenza A(H1N1)pdm09, A(H3N2) or B virus and treated with baloxavir marboxil, oseltamivir, laninamivir, or zanamivir in Japan during the 2012–2013 and 2019–2020 influenza seasons [J]. Antiviral research, 2024, 228. | Wrong population |
| [10] | Singleton J B, Wang D, Louis S, Smith D J, Lambrou A S, Beekmann S E, et al. Perceptions and Barriers to Outpatient Antiviral Therapy for COVID-19 and Influenza as Observed by Infectious Disease Specialists in North America: Results of an Emerging Infections Network (EIN) Survey, February 2024 [J]. Open Forum Infectious Diseases, 2024, 11(12). | Wrong study design |
| [11] | Shah S S, Raddawi M A R Y. A DIAGNOSTIC JOURNEY FOR PROGRESSIVE HYPOXEMIC RESPIRATORY FAILURE IN PATIENT WITH CYSTIC FIBROSIS AND LUNG TRANSPLANT [Z]. 2024: A1987-A1988.10.1016/j.chest.2024.06.1236 | Irrelevant study |
| [12] | Scherger S J, Kalil A C. In persons exposed to influenza, zanamivir, oseltamivir, laninamivir, and baloxavir reduce symptomatic seasonal influenza [J]. Annals of internal medicine, 2024. | Wrong study design |
| [13] | Sato M. Pharmacologic background and clinical issue of anti-influenza drugs [J]. Fukushima journal of medical science, 2024. | Irrelevant study |
| [14] | Sarker J, Carkovic E, Ptaszek K, Lee T A. Antiviral influenza treatments and hemorrhage-related adverse events in the United States Food and Drug Administration Adverse Event Reporting System (FAERS) database [J]. Pharmacotherapy, 2024, 44(5): 383-393. | Irrelevant study |
| [15] | Ringer M, Malinis M, McManus D, Davis M, Shah S, Trubin P, et al. Clinical outcomes of baloxavir versus oseltamivir in immunocompromised patients [J]. Transplant Infectious Disease, 2024, 26(2). | Wrong population |
| [16] | Regu V R, Swain R P, Subudhi B B. Cocrystals development: an approach to improve antiviral efficacy [J]. FUTURE VIROLOGY, 2024, 19(6-7): 225-228. | Irrelevant study |
| [17] | Raza M A, Ashraf M A. Drug resistance and possible therapeutic options against influenza A virus infection over past years [J]. ARCHIVES OF MICROBIOLOGY, 2024, 206(12). | Irrelevant study |
| [18] | Qiu C, Cheng F, Ye X, Wu Z, Ning H, Liu S, et al. Study on the clinical efficacy and safety of baloxavir marboxil tablets in the treatment of influenza A [J]. Frontiers in Medicine, 2024, 11. | Wrong comparator |
| [19] | Patel M C, Nguyen H T, Pascua P N Q, Gao R, Steel J, Kondor R J, et al. Multicountry Spread of Influenza A(H1N1)pdm09 Viruses with Reduced Oseltamivir Inhibition, May 2023-February 2024 [J]. Emerging infectious diseases, 2024, 30(7): 1410-1415. | Wrong comparator |
| [20] | Panthi S, Hong J Y, Satange R, Yu C C, Li L Y, Hou M H. Antiviral drug development by targeting RNA binding site, oligomerization and nuclear export of influenza nucleoprotein [J]. International journal of biological macromolecules, 2024, 282(Pt 4): 136996. | Irrelevant study |
| [21] | Nct. Efficacy of Baloxavir Against Influenza in Hospitalized Patients: the INFLUENT Study (INpatients InFLUENza Treatment) [J]. https://clinicaltrialsgov/ct2/show/NCT06653569, 2024. | Wrong population |
| [22] | Nakagawa N, Ono R, Odanaka K, Ohara H, Kisara S, Ito K. A Pharmacoeconomic Study of Post-Exposure Prophylaxis Strategies for Influenza Virus Infections in Japan [J]. Advances in therapy, 2024. | Irrelevant study |
| [23] | Lipka E. Phase 1 Clinical Trial to Assess the Pharmacokinetics, Safety and Tolerance of a Zanamivir Transdermal System in Healthy Subjects [J]. 2024. | Wrong comparator |
| [24] | Li Y, Wang X, Liao Y, Zeng Y, Lin W, Zhuang W. Safety analysis of Oseltamivir and Baloxavir Marboxil after market approval: a pharmacovigilance study based on the FDA adverse event reporting system [J]. BMC Infectious Diseases, 2024, 24(1). | Wrong study design |
| [25] | Li Y, Huo S, Yin Z, Tian Z, Huang F, Liu P, et al. Retracted and republished from: "The current state of research on influenza antiviral drug development: drugs in clinical trial and licensed drugs" [J]. mBio, 2024, 15(5): e0017524. | Irrelevant study |
| [26] | Kommandantvold S A, Lemenuel-Diot A, Skedgel C, Pitman R, Rouse P, Zaraket H, et al. A cost-effectiveness analysis of reduced viral transmission with baloxavir marboxil versus oseltamivir or no treatment for seasonal and pandemic influenza management in the United Kingdom [J]. Expert review of pharmacoeconomics & outcomes research, 2024, 24(8): 953-966. | Wrong study design |
| [27] | Kommandantvold S A, Chang S C, Surinach A, Yau V, Best J H, Zaraket H, et al. Cost-Effectiveness of Baloxavir Marboxil Versus Oseltamivir or no Treatment for the Management of Influenza in the United States [J]. Infectious diseases and therapy, 2024, 13(9): 2071-2087. | Wrong study design |
| [28] | Kohlbrand A J, Stokes R W, Sankaran B, Cohen S M. Structural Studies of Inhibitors with Clinically Relevant Influenza Endonuclease Variants [J]. Biochemistry, 2024, 63(3): 264-272. | Irrelevant study |
| [29] | Jiang Y, Wen J, Sun J, Shu Y. Evaluating the Public Health and Health Economic Impacts of Baloxavir Marboxil and Oseltamivir for Influenza Pandemic Control in China: A Cost-Effectiveness Analysis Using a Linked Dynamic Transmission–Economic Evaluation Model [J]. PharmacoEconomics, 2024, 42(10): 1111-1125. | Wrong study design |
| [30] | Ikematsu H, Baba T, Saito M M, Kinoshita M, Miyazawa S, Hata A, et al. Comparative Effectiveness of Baloxavir Marboxil and Oseltamivir Treatment in Reducing Household Transmission of Influenza: A Post Hoc Analysis of the BLOCKSTONE Trial [J]. Influenza and other respiratory viruses, 2024, 18(5): e13302. | Wrong outcome indicators |
| [31] | Guo X, Zhao L, Li W, Cao R, Zhong W. The Synergistic Effect of Baloxavir and Neuraminidase Inhibitors against Influenza Viruses In Vitro [J]. Viruses, 2024, 16(9). | Wrong study design |
| [32] | Guan W, Qu R, Shen L, Mai K, Pan W, Lin Z, et al. Baloxavir marboxil use for critical human infection of avian influenza A H5N6 virus [J]. Med (New York, NY), 2024, 5(1): 32-41.e35. | Irrelevant study |
| [33] | Goto T, Kawai N, Bando T, Takasaki Y, Shindo S, Tani N, et al. Virological and clinical outcomes in outpatients treated with baloxavir or neuraminidase inhibitors for A(H3N2) influenza: A multicenter study of the 2022–2023 season [J]. Antiviral research, 2024, 224. | Wrong population |
| [34] | Ringer M, Malinis M, McManus D, Davis M, Shah S, Trubin P, et al. Clinical outcomes of baloxavir versus oseltamivir in immunocompromised patients [J]. Transplant Infectious Disease, 2024, 26(2). | Wrong population |
| [35] | Gao R, Pascua P N Q, Chesnokov A, Nguyen H T, Uyeki T M, Mishin V P, et al. Antiviral Susceptibility of Swine-Origin Influenza A Viruses Isolated from Humans, United States [J]. Emerging infectious diseases, 2024, 30(11): 2303-2312. | Irrelevant study |
| [36] | Gaisina I, Li P, Du R, Cui Q, Dong M, Zhang C, et al. An orally active entry inhibitor of influenza A viruses protects mice and synergizes with oseltamivir and baloxavir marboxil [J]. Science advances, 2024, 10(8): eadk9004. | Wrong study design |
| [37] | Fortin N, Hénaut M, Goyette N, Maltais R, Sancéau J Y, Marette A, et al. A protectin DX (PDX) analog with in vitro activity against influenza A(H1N1) viruses [J]. Journal of medical virology, 2024, 96(3): e29484. | Wrong study design |
| [38] | Euzen V, Xhaard A, Berreira-Ibraim S, Deville L, Quentin A, Prata P H L, et al. Zanamivir and baloxavir combination to cure persistent influenza and coronavirus infections after hematopoietic stem cell transplant [J]. International journal of antimicrobial agents, 2024, 64(3): 107281. | Wrong comparator |
| [39] | Dockrell D H, Breen R, Collini P, Lipman M C I, Miller R F. British HIV Association guidelines on the management of opportunistic infection in people living with HIV: The clinical management of pulmonary opportunistic infections 2024 [J]. HIV Medicine, 2024, 25(S2): 3-37. | Irrelevant study |
| [40] | Din G U, Hasham K, Amjad M N, Hu Y. Natural History of Influenza B Virus—Current Knowledge on Treatment, Resistance and Therapeutic Options [J]. Current Issues in Molecular Biology, 2024, 46(1): 183-199. | Irrelevant study |
| [41] | Chon I, Win S M K, Phyu W W, Saito R, Kyaw Y, Win N C, et al. Whole-Genome Analysis of the Influenza A(H1N1)pdm09 Viruses Isolated from Influenza-like Illness Outpatients in Myanmar and Community-Acquired Oseltamivir-Resistant Strains Present from 2015 to 2019 [J]. VIRUSES-BASEL, 2024, 16(8). | Wrong study design |
| [42] | Chon I, Wagatsuma K, Saito R, Tang J W, Isamu S, Suzuki E, et al. Detection of influenza A(H3N2) viruses with polymerase acidic subunit substitutions after and prior to baloxavir marboxil treatment during the 2022–2023 influenza season in Japan [J]. Antiviral research, 2024, 229. | Wrong study design |
| [43] | Cai J, Wang H, Ye X, Lu S, Tan Z, Li Z, et al. Real-world effectiveness and safety of Baloxavir Marboxil or Oseltamivir in outpatients with uncomplicated influenza A: an ambispective, observational, multi-center study [J]. Frontiers in Microbiology, 2024, 15. | Wrong population |
| [44] | Bonomini A, Zhang J, Ju H, Zago A, Pacetti M, Tabarrini O, et al. Synergistic activity of an RNA polymerase PA-PB1 interaction inhibitor with oseltamivir against human and avian influenza viruses in cell culture and <i>in ovo</i> [J]. Antiviral research, 2024, 230. | Irrelevant study |
| [45] | Bleik P, Zakharchenko E G O R. ACUTE RESPIRATORY DISTRESS SYNDROME IN PATIENT WITH TREATMENT-RESISTANT INFLUENZA THAT REQUIRED MANAGEMENT WITH BALOXAVIR [Z]. 2024: A1872-A1873.10.1016/j.chest.2024.06.1173 | Wrong study design |
| [46] | Best J H, Sadeghi M, Sun X, Seetasith A, Albensi L, Joshi S, et al. Household Influenza Transmission and Healthcare Resource Utilization Among Patients Treated with Baloxavir vs Oseltamivir: A United States Outpatient Prospective Survey [J]. Infectious diseases and therapy, 2024. | Wrong study design |
| [47] | Bassetti M, Sepulcri C, Giacobbe D R, Fusco L. Treating influenza with neuraminidase inhibitors: an update of the literature [J]. Expert opinion on pharmacotherapy, 2024, 25(9): 1163-1174. | Wrong study design |
| [48] | Barbin G. Fingerprint of the Most Prevalent Respiratory Viral Strains on In Vitro Primary Human Nasal Epithelium [J]. American journal of respiratory and critical care medicine, 2024, 209. | Irrelevant study |
| [49] | Azuma T, Usui M, Hasei T, Hayashi T. Occurrence and environmental fate of anti-influenza drugs in a subcatchment of the Yodo River Basin, Japan [J]. Science of the Total Environment, 2024, 953. | Irrelevant study |
| [50] | Ashtiwi N M Z. The Therapeutic Potential of Thiocyanate Administration in Influenza Lung Infection [M]. 2024. | Irrelevant study |
| [51] | Andreev K, Jones J C, Seiler P, Kandeil A, Webby R J, Govorkova E A. Genotypic and phenotypic susceptibility of emerging avian influenza A viruses to neuraminidase and cap-dependent endonuclease inhibitors [J]. Antiviral research, 2024, 229: 105959. | Irrelevant study |
| [52] | Andreev K, Jones J C, Seiler P, Kandeil A, Turner J C M, Barman S, et al. Antiviral Susceptibility of Highly Pathogenic Avian Influenza A(H5N1) Viruses Circulating Globally in 2022–2023 [J]. Journal of Infectious Diseases, 2024, 229(6): 1830-1835. | Irrelevant study |
| [53] | Erratum: Targeted recruitment of immune effector cells for rapid eradication of influenza virus infections (Proceedings of the National Academy of Sciences of the United States of America (2024) 121 (e2408469121) DOI: 10.1073/pnas.2408469121) [J]. Proceedings of the National Academy of Sciences of the United States of America, 2024, 121(49). | Irrelevant study |
| [54] | Liu Bo, Li Suying. Analysis of Influenza A Infection and Treatment Status in a University in Beijing during the Influenza Epidemic Season in 2023 [J]. 2023, 30 J International Journal of Virology. | Wrong study design |
| [55] | Gan Kao, Cheng Ye, Chen Yun, Tao Lanting, Li Fang, Chen Lixia. A Retrospective Study on the Treatment of Influenza A with Traditional Chinese Medicine Combined with Anti-influenza Virus Drugs J Journal of Changchun University of Chinese Medicine [J]. 2023, 39(02): 171-174. | Wrong study design |
| [56] | Cai Peishan. What are the Differences between the New Anti-influenza Drug Marboxil and Oseltamivir? J Medicine & People [J]. 2023, (3): 40-43. | Irrelevant study |
| [57] | Westin J, Andersson E, Bengnér M, Berggren A, Brytting M, Ginström Ernstad E, et al. Management of influenza–updated Swedish guidelines for antiviral treatment [J]. Infectious Diseases, 2023, 55(10): 725-737. | Irrelevant study |
| [58] | Tenforde M W, Cummings C N, O'Halloran A C, Rothrock G, Kirley P D, Alden N B, et al. Influenza Antiviral Use in Patients Hospitalized With Laboratory-Confirmed Influenza in the United States, FluSurv-NET, 2015-2019 [J]. Open Forum Infectious Diseases, 2023, 10(1). | Irrelevant study |
| [59] | Takashita E, Fujisaki S, Morita H, Nagata S, Miura H, Matsuura Y, et al. A community cluster of influenza A(H3N2) virus infection with reduced susceptibility to baloxavir due to a PA E199G substitution in Japan, February to March 2023 [J]. Eurosurveillance, 2023, 28(39). | Wrong study design |
| [60] | Soga T, Duong C, Pattinson D, Sakai-Tagawa Y, Tokita A, Izumida N, et al. Characterization of Influenza A(H1N1)pdm09 Viruses Isolated in the 2018–2019 and 2019–2020 Influenza Seasons in Japan [J]. Viruses, 2023, 15(2). | Wrong study design |
| [61] | Singh P P, Sodhi K K, Bali A K, Shree P. Influenza A virus and its antiviral drug treatment options [J]. Medicine in Microecology, 2023, 16. | Irrelevant study |
| [62] | Shea L, Goldwire M A. Navigating Common Upper Respiratory Tract Conditions [Z]. 2023: 46-56 | Irrelevant study |
| [63] | Shahriar I, Campbell C L, Kanduluru A K, Srinivasarao M, Low P S. Design of a Ligand-Targeted Immunotherapy for Treatment of Influenza Virus Infections [J]. Open Forum Infectious Diseases, 2023, 10: S906. | Irrelevant study |
| [64] | Sato M, Takashita E, Katayose M, Nemoto K, Sakai N, Fujisaki S, et al. Clinical and Virologic Impacts of Respiratory Viral Co-infections in Children With Influenza [J]. The Pediatric infectious disease journal, 2023, 42(8): e268-e273. | Irrelevant study |
| [65] | Sako A, Gu Y, Masui Y, Yoshimura K, Yanai H, Ohmagari N. Prescription of anti-influenza drugs in Japan, 2014-2020: A retrospective study using open data from the national claims database [J]. PloS one, 2023, 18(10): e0291673-e0291673. | Wrong study design |
| [66] | Saim-Mamoun A, Carbonneau J, Rheaume C, Abed Y, Boivin G. Viral Fitness of Baloxavir-Resistant Recombinant Influenza B/Victoria- and B/Yamagata-like Viruses Harboring the I38T PA Change, In Vitro, Ex Vivo and in Guinea Pigs [J]. Microorganisms, 2023, 11(5). | Wrong study design |
| [67] | Rong L. Development of 4-(aroylamino)piperidine-based entry inhibitors as anti-influenza therapeutics [J]. 2023. | Irrelevant study |
| [68] | Rogers J H, Casto A M, Nwanne G, Link A C, Martinez M A, Nackviseth C, et al. Results from a test-and-treat study for influenza among residents of homeless shelters in King County, WA: A stepped-wedge cluster-randomized trial [J]. Influenza and other respiratory viruses, 2023, 17(1): e13092. | Irrelevant study |
| [69] | Ou G, Xu H, Wu J, Wang S, Chen Y, Deng L, et al. The gut-lung axis in influenza A: the role of gut microbiota in immune balance [J]. Frontiers in immunology, 2023, 14: 1147724. | Irrelevant study |
| [70] | Okubo Y, Uda K, Miyairi I. yy Trends in Influenza and Related Health Resource Use During 2005-2021 Among Children in Japan [J]. PEDIATRIC INFECTIOUS DISEASE JOURNAL, 2023, 42(8): 648-653. | Wrong study design |
| [71] | Okubo Y, Uda K, Miyairi I. Trends in Influenza and Related Health Resource Use during 2005-2021 among Children in Japan [J]. Pediatric Infectious Disease Journal, 2023, 42(8): 648-653. | Wrong study design |
| [72] | Oh D-Y, Milde J, Ham Y, Calderon J P R, Wedde M, Duerrwald R, et al. Preparing for the Next Influenza Season: Monitoring the Emergence and Spread of Antiviral Resistance [J]. Infection and drug resistance, 2023, 16: 949-959. | Wrong study design |
| [73] | Nguyen H T, Chesnokov A, De La Cruz J, Pascua P N Q, Mishin V P, Jang Y, et al. Antiviral susceptibility of clade 2.3.4.4b highly pathogenic avian influenza A(H5N1) viruses isolated from birds and mammals in the United States, 2022 [J]. Antiviral research, 2023, 217: 105679. | Irrelevant study |
| [74] | Nezu K, Hayashida S, Nagano N, Udagawa S, Morioka I. Early Fever Resolution in Early Childhood Influenza Treated with Baloxavir Marboxil: A Retrospective Study Compared to Those with Oseltamivir [J]. Medicina (Kaunas, Lithuania), 2023, 59(9). | Low-quality study |
| [75] | Murray J, Martin D E, Sancilio F D, Tripp R A. Antiviral Activity of Probenecid and Oseltamivir on Influenza Virus Replication [J]. Viruses, 2023, 15(12). | Wrong comparator |
| [76] | Meseko C, Sanicas M, Asha K, Sulaiman L, Kumar B. Antiviral options and therapeutics against influenza: history, latest developments and future prospects [J]. Frontiers in cellular and infection microbiology, 2023, 13. | Irrelevant study |
| [77] | Marquez-Dominguez L, Marquez-Matla K, Reyes-Leyva J, Vallejo-Ruiz V, Santos-Lopez G. Antiviral resistance in influenza viruses [J]. CELLULAR AND MOLECULAR BIOLOGY, 2023, 69(13): 16-23. | Irrelevant study |
| [78] | Luo J, Zhang Z, Zhao S, Gao R. A Comparison of Etiology, Pathogenesis, Vaccinal and Antiviral Drug Development between Influenza and COVID-19 [J]. International journal of molecular sciences, 2023, 24(7). | Irrelevant study |
| [79] | Luo D, Ye Q, Li R T, Zhou H Y, Guo J J, Zhao S Q, et al. PA-E18G substitution in influenza A virus confers resistance to ZX-7101, a cap-dependent endonuclease inhibitor [J]. Virologica Sinica, 2023, 38(4): 559-567. | Irrelevant study |
| [80] | Liao G, Xia M, Jiang Y, Chen H, Liao W, Peng J, et al. Prospective observational study of baloxavir marboxil in adults and adolescents with uncomplicated influenza from China [J]. Frontiers in Microbiology, 2023, 14. | Wrong population |
| [81] | Liang S. Antiviral drugs against influenza viruses: Oseltamivir and baloxavir [J]. Proceedings of SPIE, 2023: 1261161 (1261112 pp.)-1261161 (1261112 pp.). | Wrong research type |
| [82] | Li Y, Huo S, Yin Z, Tian Z, Huang F, Liu P, et al. The current state of research on influenza antiviral drug development: drugs in clinical trial and licensed drugs [J]. mBio, 2023, 14(5): e0127323. | Wrong study design |
| [83] | Li J, Wagatsuma K, Sun Y, Sato I, Kawashima T, Saito T, et al. Factors associated with viral RNA shedding and evaluation of potential viral infectivity at returning to school in influenza outpatients after treatment with baloxavir marboxil and neuraminidase inhibitors during 2013/2014–2019/2020 seasons in Japan: an observational study [J]. BMC Infectious Diseases, 2023, 23(1). | Wrong study design |
| [84] | Levin J, Döhrmann S, Cole J N, Amundson K, Borchardt A, Brady T P, et al. 2062. In Vivo Efficacy of CD388, a Novel Drug Fc-Conjugate (DFC), Against Seasonal Subtypes of Influenza in Prophylaxis in Immune Competent Mice, and in a Severe Immunodeficient (SCID) Mouse Model [J]. Open Forum Infectious Diseases, 2023, 10: S79-S80. | Wrong study design |
| [85] | Kuroda T, Fukao K, Yoshida S, Oka R, Baba K, Ando Y, et al. In Vivo Antiviral Activity of Baloxavir against PA/I38T-Substituted Influenza A Viruses at Clinically Relevant Doses [J]. Viruses, 2023, 15(5). | Wrong study design |
| [86] | Kiso M, Yamayoshi S, Kawaoka Y. Efficacy of favipiravir against influenza virus resistant to both baloxavir and neuraminidase inhibitors [J]. The Journal of antimicrobial chemotherapy, 2023, 78(7): 1649-1657. | Wrong comparator |
| [87] | Kandeil A, Patton C, Jones J C, Jeevan T, Harrington W N, Trifkovic S, et al. Rapid evolution of A(H5N1) influenza viruses after intercontinental spread to North America [J]. Nature communications, 2023, 14(1). | Wrong research type |
| [88] | Jones J C, Yen H-L, Adams P, Armstrong K, Govorkova E A. Influenza antivirals and their role in pandemic preparedness [J]. Antiviral research, 2023, 210. | Wrong research type |
| [89] | Hirotsu N, Sakaguchi H, Fukao K, Kojima S, Piedra P A, Tsuchiya K, et al. Baloxavir safety and clinical and virologic outcomes in influenza virus-infected pediatric patients by age group: age-based pooled analysis of two pediatric studies conducted in Japan [J]. BMC PEDIATRICS, 2023, 23(1). | Low-quality study |
| [90] | Hickerson B T, Huang B K, Petrovskaya S N, Ilyushina N A. Genomic Analysis of Influenza A and B Viruses Carrying Baloxavir Resistance-Associated Substitutions Serially Passaged in Human Epithelial Cells [J]. VIRUSES-BASEL, 2023, 15(12). | Wrong study design |
| [91] | Hansen S, Cheng S C, Surinach A, Yau V, Best J H, Zaraket H, et al. Cost-effectiveness of baloxavir marboxil versus oseltamivir or no treatment for the management of influenza in the United States [J]. Open Forum Infectious Diseases, 2023, 10: S502. | Wrong study design |
| [92] | Guan W, Qu R, Shen L, Mai K, Pan W, Lin Z, et al. Baloxavir marboxil use for critical human infection of avian influenza A H5N6 virus [J]. medRxiv, 2023. | Wrong research type |
| [93] | Guan W, Qu R, Shen L, Mai K, Pan W, Lin Z, et al. Baloxavir marboxil use for critical human infection of avian influenza A H5N6 virus [Z]. 2023.10.1101/2023.09.03.23294799 | Wrong research type |
| [94] | Fukao K, Noshi T, Shano S, Baba K, Sato K, Sakuramoto M, et al. Prophylactic Treatment with Baloxavir Protects Mice from Lethal Infection with Influenza A and B Viruses [J]. Viruses, 2023, 15(11). | Wrong study design |
| [95] | Fujiwara N, Fujiwara T, Ise Y. Prescription factors influencing baloxavir prescription during the 2018/2019 and 2019/2020 seasons: a administrative database study in Japan [J]. Journal of pharmaceutical health care and sciences, 2023, 9(1). | Wrong study design |
| [96] | Earnhardt E Y, Tipper J L, D’Mello A, Jian M Y, Conway E S, Mobley J A, et al. Influenza A Induced Cystic Fibrosis Transmembrane Conductance Regulator Dysfunction Increases Susceptibility to Streptococcus pneumoniae [J]. JCI Insight, 2023, 8(14). | Irrelevant study |
| [97] | Donadello K, Gottin L, Boetti R P, Martin-Loeches I. Influenza management with new therapies [J]. Minerva Respiratory Medicine, 2023, 62(3): 135-145. | Irrelevant study |
| [98] | Cruz C D, Icochea M E, Espejo V, Troncos G, Castro-Sanguinetti G R, Schilling M A, et al. Highly Pathogenic Avian Influenza A(H5N1) from Wild Birds, Poultry, and Mammals, Peru [J]. Emerging infectious diseases, 2023, 29(12): 2572-2576. | Irrelevant study |
| [99] | Crunkhorn S. Inhibiting cap snatching [J]. NATURE REVIEWS DRUG DISCOVERY, 2023, 22(4): 271-271. | Irrelevant study |
| [100] | Collins C, Cagas S E, Han J, Delporte M L, Retout S. Baloxavir Marboxil (BXM) Treatment of Influenza in Renally Impaired Patients: Post Hoc Analysis of CAPSTONE-2 [J]. Journal of the American Society of Nephrology, 2023, 34: 392. | Wrong population |
| [101] | Clercq E D. Management of viral infections: vaccines or antivirals [J]. FUTURE VIROLOGY, 2023, 18(16). | Irrelevant study |
| [102] | Chen X, Ma Q, Zhao M, Yao Y, Zhang Q, Liu M, et al. Preclinical Study of ZSP1273, a Potent Antiviral Inhibitor of Cap Binding to the PB2 Subunit of Influenza A Polymerase [J]. Pharmaceuticals, 2023, 16(3). | Irrelevant study |
| [103] | Chan K K P, Hui D S C. Antiviral therapies for influenza [J]. Current opinion in infectious diseases, 2023, 36(2): 124-131. | Irrelevant study |
| [104] | Bulloch M N. Treatment and prevention of influenza in geriatric patients [J]. Expert Review of Clinical Pharmacology, 2023, 16(9): 825-841. | Irrelevant study |
| [105] | Best J H, Sadeghi M H, Sun X, Seetasith A H, Albensi L, Joshi S, et al. Household Flu Transmission and Healthcare Resource Use among Patients Treated with Baloxavir versus Oseltamivir for Influenza: An Outpatient Prospective Survey in the United States [J]. Open Forum Infectious Diseases, 2023, 10: S500-S501. | Wrong study design |
| [106] | Best J, Brady B, Park J, Larkin H, Collins C, Seetasith A. EE127 Healthcare Resource Utilization Among Influenza Patients Treated with Baloxavir Marboxil Compared with Oseltamivir with Medicaid Insurance Coverage [J]. Value in Health, 2023, 26(6): S82. | Wrong study design |
| [107] | Barnes A, Ringer M, Malinis M, McManus D, Davis M W, Shah S, et al. Clinical Outcomes of Baloxavir vs. Oseltamivir in Transplant and Other Immunocompromised Patients [J]. American Journal of Transplantation, 2023, 23(6): S1110. | Wrong population |
| [108] | Barnes A, Ringer M, Malinis M, McManus D, Davis M W, Shah S, et al. Clinical Outcomes of Baloxavir vs. Oseltamivir in Transplant and Other Immunocompromised Patients [J]. American Journal of Transplantation, 2023, 23(6): S1110. | Wrong population |
| [109] | Asher J, Lemenuel-Diot A, Clay M, Durham D P, Mier-Y-Teran-Romero L, Arguello C J, et al. Novel modelling approaches to predict the role of antivirals in reducing influenza transmission [J]. PLoS Computational Biology, 2023, 19(1). | Irrelevant study |
| [110] | Antoon J W, Sarker J, Abdelaziz A, Lien P-W, Williams D J, Lee T A, et al. Trends in Outpatient Influenza Antiviral Use Among Children and Adolescents in the United States [J]. Pediatrics, 2023, 152(6). | Irrelevant study |
| [111] | Antoon J W, Sarker J, Abdelaziz A, Lien P W, Williams D J, Lee T A, et al. Trends in Outpatient Influenza Antiviral Use Among Children and Adolescents in the United States [J]. Pediatrics, 2023, 152(6). | Irrelevant study |
| [112] | Alqaissi E, Alotaibi F, Sher Ramzan M, Algarni A. Novel graph-based machine-learning technique for viral infectious diseases: application to influenza and hepatitis diseases [J]. Annals of medicine, 2023, 55(2). | Irrelevant study |
| [113] | Al-Azzawi S, Masheta D. Impact of the COVID-19 pandemic on dispensing medicines in the community pharmacy [J]. International Journal of Risk and Safety in Medicine, 2023, 34(4): 295-311. | Irrelevant study |
| [114] | Alasiri A, Soltane R, Hegazy A, Khalil A M, Mahmoud S H, Khalil A A, et al. Vaccination and Antiviral Treatment against Avian Influenza H5Nx Viruses: A Harbinger of Virus Control or Evolution [J]. Vaccines, 2023, 11(11). | Irrelevant study |
| [115] | Abdul Y, Davidson E, McConeghy K, Hayes K, Han L, LaMantia M, et al. The Impact of Cost-Free On-Site Influenza Point of Care Antigen Testing on Influenza Detection in Nursing Homes [J]. Open Forum Infectious Diseases, 2023, 10: S491-S492. | Irrelevant study |
| [116] | The Medical Letter® on Drugs and Therapeutics [J]. Medical Letter on Drugs and Therapeutics, 2023, 65(1687): 161-166. | Irrelevant study |
| [117] | Antiviral Drugs for Influenza for 2023-2024 [J]. Medical Letter on Drugs and Therapeutics, 2023, 165(1689): 177-182. | Irrelevant study |
| [118] | Weill Medical College of Cornell U. Baloxavir in Combination With Oseltamivir in Allogenic Bone Marrow Transplant Recipients With Influenza [J]. ClinicalTrialsgov, 2022. | Wrong study design |
| [119] | Taniguchi K, Noshi T, Omoto S, Sato A, Shishido T, Matsuno K, et al. The impact of PA/I38 substitutions and PA polymorphisms on the susceptibility of zoonotic influenza A viruses to baloxavir [J]. Archives of virology, 2024, 169(2): 29. | Wrong study design |
| [120] | Singleton J B, Wang D, Louis S, Smith D J, Lambrou A S, Beekmann S E, et al. Perceptions and Barriers to Outpatient Antiviral Therapy for COVID-19 and Influenza as Observed by Infectious Disease Specialists in North America: Results of an Emerging Infections Network (EIN) Survey, February 2024 [J]. Open Forum Infectious Diseases, 2024, 11(12). | Irrelevant study |
| [121] | Varghese P M, Kishore U, Rajkumari R. Innate and adaptive immune responses against Influenza A Virus: Immune evasion and vaccination strategies [J]. Immunobiology, 2022, 227(6). | Irrelevant study |
| [122] | Terrie Y C. Antiviral Recommendations for the 2022–2023 Influenza Season [Z]. 2022: 37-42 | Irrelevant study |
| [123] | Tejus A, Mathur A G, Pradhan S, Malik S, Salmani M F. Drug update - Baloxavir marboxil: Latest entrant into the arena of pharmacotherapy of influenza [J]. Medical journal, Armed Forces India, 2022, 78(2): 125-130. | Wrong research type |
| [124] | Taniguchi K, Ando Y, Kobayashi M, Toba S, Nobori H, Sanaki T, et al. Characterization of the In Vitro and In Vivo Efficacy of Baloxavir Marboxil against H5 Highly Pathogenic Avian Influenza Virus Infection [J]. Viruses, 2022, 14(1). | Wrong study design |
| [125] | Tani N, Kawai N, Chong Y, Bando T, Iwaki N, Kashiwagi S, et al. Susceptibility of epidemic viruses to neuraminidase inhibitors and treatment-emergent resistance in the Japanese 2019-20 influenza season [J]. Journal of Infection, 2022, 84(2): 151-157. | Wrong study design |
| [126] | Tang Y, Xia Y, Tang M, Jiang H. POSB163 Cost-Effectiveness Analysis of Baloxavir Barboxil As Seasonal Influenza Treatment Compared to Oseltamivir in China [J]. Value in Health, 2022, 25(1): S92. | Wrong study design |
| [127] | Takizawa N, Takada H, Umekita M, Igarashi M, Takahashi Y. Anti-influenza Virus Activity of Methylthio-Formycin Distinct From That of T-705 [J]. Frontiers in Microbiology, 2022, 13. | Irrelevant study |
| [128] | Takashita E, Morita H, Nagata S, Shirakura M, Fujisaki S, Miura H, et al. Antiviral Susceptibilities of Avian Influenza A(H5), A(H7), and A(H9) Viruses Isolated in Japan [J]. Japanese journal of infectious diseases, 2022, 75(4): 398-402. | Irrelevant study |
| [129] | Swierczynska M, Mirowska-Guzel D M, Pindelska E. Antiviral Drugs in Influenza [J]. International journal of environmental research and public health, 2022, 19(5). | Irrelevant study |
| [130] | Stannard H L, Mifsud E J, Wildum S, Brown S K, Koszalka P, Shishido T, et al. Assessing the fitness of a dual-antiviral drug resistant human influenza virus in the ferret model [J]. COMMUNICATIONS BIOLOGY, 2022, 5(1). | Irrelevant study |
| [131] | Sridhar S, To K K W. Severe influenza: is there a role for antiviral combinations? [J]. The Lancet Infectious Diseases, 2022, 22(5): 574-576. | Irrelevant study |
| [132] | Sousa T d C, Martins J S C C, Miranda M D, Garcia C C, Resende P C, Santos C A A, et al. Low prevalence of influenza A strains with resistance markers in Brazil during 2017-2019 seasons [J]. Frontiers in public health, 2022, 10. | Irrelevant study |
| [133] | Smyk J M, Szydłowska N, Szulc W, Majewska A. Evolution of Influenza Viruses—Drug Resistance, Treatment Options, and Prospects [J]. International journal of molecular sciences, 2022, 23(20). | Irrelevant study |
| [134] | Shinno K, Takeuchi M, Kawakami K. Association between Baloxavir Marboxil Prescription for Children with Influenza B Infections and Short-Term Healthcare Consumption in Japan during the 2018-2019 Influenza Season [J]. Journal of the Pediatric Infectious Diseases Society, 2022, 11(7): 310-315. | Wrong outcome |
| [135] | Shimizu E, Iwasaki K, Hongo Y, Yoshida M, Kinoshita M, Hiroi S, et al. Diagnosis and treatment of influenza based on health insurance claims between the 2010–2011 and 2019–2020 influenza seasons in Japan [J]. Influenza and other respiratory viruses, 2022, 16(4): 621-625. | Wrong study design |
| [136] | Seldeslachts L, Jacobs C, Tielemans B, Vanhoffelen E, Van der Sloten L, Humblet-Baron S, et al. Overcome Double Trouble: Baloxavir Marboxil Suppresses Influenza Thereby Mitigating Secondary Invasive Pulmonary Aspergillosis [J]. JOURNAL OF FUNGI, 2022, 8(1). | Irrelevant study |
| [137] | Sarker J, Abdelaziz A, Lien P W, Grijalva C, Williams D, Lee T A, et al. Trends in antiviral use for influenza among children in the US between 2010 and 2019 [J]. Pharmacoepidemiology and drug safety, 2022, 31: 576. | Irrelevant study |
| [138] | Sarker A, Gu Z, Mao L, Ge Y, Hou D, Fang J, et al. Influenza-existing drugs and treatment prospects [J]. European journal of medicinal chemistry, 2022, 232. | Irrelevant study |
| [139] | Sako A, Gu Y, Masui Y, Yoshimura K, Yanai H, Ohmagari N. Prescription of anti-influenza drugs in Japan, 2014-2020: a retrospective study using open data from the national claims database [Z]. 2022.10.1101/2022.11.15.22281290 | Wrong study design |
| [140] | Saim-Mamoun A, Abed Y, Carbonneau J, Boivin G. Generation and Characterization of Drug-Resistant Influenza B Viruses Selected In Vitro with Baloxavir Acid [J]. Pathogens (Basel, Switzerland), 2022, 11(9). | Wrong study design |
| [141] | Rong L. Development of 4-(aroylamino)piperidine-based entry inhibitors as anti-influenza therapeutics [J]. 2022. | Irrelevant study |
| [142] | Qin J, Lin J, Zhang X, Yuan S, Zhang C, Yin Y. Evaluation of the Clinical Effectiveness of Oseltamivir for Influenza Treatment in Children [J]. Frontiers in pharmacology, 2022, 13. | Wrong comparator |
| [143] | Patel M C, Flanigan D, Feng C, Chesnokov A, Nguyen H T, Elal A A, et al. An optimized cell-based assay to assess influenza virus replication by measuring neuraminidase activity and its applications for virological surveillance [J]. Antiviral research, 2022, 208: 105457. | Irrelevant study |
| [144] | Pandey P, Karupiah G. Targeting tumour necrosis factor to ameliorate viral pneumonia [J]. FEBS Journal, 2022, 289(4): 883-900. | Irrelevant study |
| [145] | Oriola A O, Oyedeji A O. Essential Oils and Their Compounds as Potential Anti-Influenza Agents [J]. Molecules (Basel, Switzerland), 2022, 27(22). | Irrelevant study |
| [146] | O'Leary S T, Campbell J D, Ardura M I, Banerjee R, Bryant K A, Caserta M T, et al. Recommendations for Prevention and Control of Influenza in Children, 2022-2023 [J]. Pediatrics, 2022, 150(4). | Wrong study design |
| [147] | Neuberger E, Wallick C, Chawla D, de Cassia Castro R. Baloxavir vs Oseltamivir: Reduced Utilization and Costs in Influenza [J]. American Journal of Managed Care, 2022, 28(3): E88-E95. | Wrong outcome |
| [148] | Motisi M A, Tamborino A, Parigi S, Galli L, de Martino M, Chiappini E. The use of antiviral drugs in children [J]. Journal of Chemotherapy, 2022, 34(2): 73-86. | Wrong research type |
| [149] | Miyazawa S, Takazono T, Hosogaya N, Yamamoto K, Watanabe H, Fujiwara M, et al. Comparison of Intra-Familial Transmission of Influenza Virus From Index Patients Treated With Baloxavir Marboxil or Oseltamivir Using an Influenza Transmission Model and a Health Insurance Claims Database [J]. Clinical infectious diseases : an official publication of the Infectious Diseases Society of America, 2022, 75(6): 927-935. | Wrong study design |
| [150] | Mikamo H, Koizumi Y, Yamagishi Y, Asai N, Miyazono Y, Shinbo T, et al. Comparing the cobas Influenza A/B Nucleic acid test for use on the cobas Liat System (Liat) with rapid antigen tests for clinical management of Japanese patients at the point of care [J]. PloS one, 2022, 17(10 October). | Wrong study design |
| [151] | Lampejo T. Is combination antiviral therapy for influenza the optimal approach? [J]. The Lancet Infectious Diseases, 2022, 22(5): 587-588. | Wrong research type |
| [152] | Kurazono K, Ikeoka H, Hiroi S, Iwasaki K, Takeshima T, Akazawa M. Estimation of the cost of influenza antiviral medication guidance or support provided by healthcare professionals: a questionnaire survey in Japan [J]. Journal of Medical Economics, 2022, 25(1): 38-50. | Wrong study design |
| [153] | Kumar D, Ison M G, Mira J-P, Welte T, Ha J H, Hui D S, et al. Combining baloxavir marboxil with standard-of-care neuraminidase inhibitor in patients hospitalised with severe influenza (FLAGSTONE): a randomised, parallel-group, double-blind, placebo-controlled, superiority trial [J]. LANCET INFECTIOUS DISEASES, 2022, 22(5): 718-730. | Wrong comparator |
| [154] | Koszalka P, Subbarao K, Baz M. Preclinical and clinical developments for combination treatment of influenza [J]. PLoS pathogens, 2022, 18(5). | Irrelevant study |
| [155] | Koszalka P, George A, Dhanasekaran V, Hurt A C, Subbarao K. Effect of Baloxavir and Oseltamivir in Combination on Infection with Influenza Viruses with PA/I38T or PA/E23K Substitutions in the Ferret Model [J]. mBio, 2022, 13(4): e0105622. | Wrong study design |
| [156] | Kalai T, Pongracz J E, Matyus P. Recent Advances in Influenza, HIV and SARS-CoV-2 Infection Prevention and Drug Treatment-The Need for Precision Medicine [J]. CHEMISTRY-SWITZERLAND, 2022, 4(2): 216-258. | Wrong comparator |
| [157] | Kakuya F, Okubo H, Fujiyasu H, Kurisawa M J, Kinebuchi T. Clinical effectiveness of baloxavir marboxil against influenza in three seasons [J]. Pediatrics international : official journal of the Japan Pediatric Society, 2022, 64(1): e15169. | Irrelevant study |
| [158] | Juárez-Méndez M T, Borges-Argáez R, Ayora-Talavera G, Escalante-Rebolledo S E, Escalante-Erosa F, Cáceres-Farfán M. Diospyros anisandra phytochemical analysis and anti-hemagglutinin-neuraminidase activity on influenza AH1N1pdm09 virus [J]. Natural product research, 2022, 36(10): 2666-2672. | Wrong comparator |
| [159] | Jiang Y, Lin Y-F, Shi S, Chen D, Shu Y. Effects of baloxavir and oseltamivir antiviral therapy on the transmission of seasonal influenza in China: A mathematical modeling analysis [J]. Journal of medical virology, 2022, 94(11): 5425-5433. | Wrong study design |
| [160] | Ison M G. Respiratory viral infections in the immunocompromised [J]. Current opinion in pulmonary medicine, 2022, 28(3): 205-210. | Irrelevant study |
| [161] | Hickerson B T, Adams S E, Barman S, Miller L, Lugovtsev V Y, Webby R J, et al. Pleiotropic Effects of Influenza H1, H3, and B Baloxavir-Resistant Substitutions on Replication, Sensitivity to Baloxavir, and Interferon Expression [J]. Antimicrobial agents and chemotherapy, 2022, 66(4). | Wrong outcome |
| [162] | Hayden F G, Asher J, Cowling B J, Hurt A C, Ikematsu H, Kuhlbusch K, et al. Reducing Influenza Virus Transmission: The Potential Value of Antiviral Treatment [J]. CLINICAL INFECTIOUS DISEASES, 2022, 74(3): 532-540. | Irrelevant study |
| [163] | Hara A, Hara K, Komeda T, Ogura E, Miyazawa S, Kobayashi C, et al. Comparison of the incidence of bleeding between baloxavir marboxil and other anti-influenza drugs among outpatients with influenza virus infection: A retrospective cohort study using an employment-based health insurance claims database in Japan [J]. Pharmacoepidemiology and drug safety, 2022, 31(6): 623-631. | Wrong comparator |
| [164] | Govorkova E A, Takashita E, Daniels R S, Fujisaki S, Presser L D, Patel M C, et al. Global update on the susceptibilities of human influenza viruses to neuraminidase inhibitors and the cap-dependent endonuclease inhibitor baloxavir, 2018-2020 [J]. Antiviral research, 2022, 200: 105281. | Irrelevant study |
| [165] | Eichberg J, Maiworm E, Oberpaul M, Czudai-Matwich V, Lüddecke T, Vilcinskas A, et al. Antiviral Potential of Natural Resources against Influenza Virus Infections [J]. Viruses, 2022, 14(11). | Irrelevant study |
| [166] | Dufrasne F. Baloxavir Marboxil: An Original New Drug against Influenza [J]. PHARMACEUTICALS, 2022, 15(1). | Wrong research type |
| [167] | Collins C, Han J, Cagas S E, Stephens J, Sun C, Kuhlbusch K. BALOXAVIR MARBOXIL FOR THE TREATMENT OF INFLUENZA VIRUS INFECTION IN PATIENTS WITH CARDIOVASCULAR DISEASE: A SUBGROUP ANALYSIS OF CAPSTONE-2 [J]. Journal of the American College of Cardiology, 2022, 79(9): 1632. | Wrong comparator |
| [168] | Chauhan R P, Gordon M L. An overview of influenza A virus genes, protein functions, and replication cycle highlighting important updates [J]. Virus genes, 2022, 58(4): 255-269. | Irrelevant study |
| [169] | Cagas S, Gupta S, Han J, McIntosh M, Collins C, Sun C, et al. Efficacy and Safety of Baloxavir Marboxil for the Treatment of Influenza Virus Infection in Patients with Chronic Lung Disease: A Subgroup Analysis of CAPSTONE-2 [J]. American journal of respiratory and critical care medicine, 2022, 205(1). | Wrong population |
| [170] | Caceres C J, Seibert B, Faccin F C, Cardenas-Garcia S, Rajao D S, Perez D R. Influenza antivirals and animal models [J]. FEBS open bio, 2022, 12(6): 1142-1165. | Irrelevant study |
| [171] | Caceres C J, Seibert B, Cargnin Faccin F, Cardenas-Garcia S, Rajao D S, Perez D R. Influenza antivirals and animal models [J]. FEBS open bio, 2022, 12(6): 1142-1165. | Irrelevant study |
| [172] | Brehm T T, Hennigs A. Seasonal influenza - updates on epidemiology, prevention and therapy [J]. DEUTSCHE MEDIZINISCHE WOCHENSCHRIFT, 2022, 147(22): 1456-1463. | Irrelevant study |
| [173] | Best J, Seetasith A, Reddy S, Chang E, Bognar K, Tarbox M, et al. Mortality and economic burden of seasonal influenza among elderly Medicare beneficiaries with and without antiviral treatment [J]. Journal of Managed Care and Specialty Pharmacy, 2022, 28(10): S83-S84. | Irrelevant study |
| [174] | Best J, Park J, Larkin H, Brady B, Sedeghi M, Seetasith A. Healthcare resource utilization among influenza patients treated with baloxavir marboxil compared with oseltamivir [J]. Journal of Managed Care and Specialty Pharmacy, 2022, 28(10): S83. | Irrelevant study |
| [175] | Benitez A, Xu H, Ukachukwu V, Chawla D. Treatment of Influenza with Baloxavir was Associated with Reduced Absenteeism Compared with Oseltamivir in a Patient-Generated Health Data Study [J]. Open Forum Infectious Diseases, 2022, 9: S641. | Wrong intervention |
| [176] | Aoki F Y, Papenburg J, Mubareka S, Allen U D, Hatchette T F, Evans G A. 2021–2022 AMMI Canada guidance on the use of antiviral drugs for influenza in the COVID-19 pandemic setting in Canada [J]. JAMMI, 2022, 7(1): 1-7. | Irrelevant study |
| [177] | Antoon J, Hall M, Feinstein J, Kyler K, Shah S, Girdwood S T, et al. Prevalence and factors associated with guideline concordant antiviral treatment in children at high risk for influenza complications [J]. Pharmacoepidemiology and drug safety, 2022, 31: 572-573. | Irrelevant study |
| [178] | Angelidakis G, Khawaja F, Mulanovich V E, Dailey-Garnes N, Ariza-Heredia E, Chemaly R F. Combination of baloxavir and oseltamivir for treatment of severe influenza infection in hematopoietic cell transplant recipients: a novel treatment strategy for a high-risk population [J]. Microbes and Infection, 2022, 24(3). | Wrong population |
| [179] | Antiviral Drugs for Influenza for 2021-2022 [J]. Medical Letter on Drugs and Therapeutics, 2022, 64(1641). | Irrelevant study |
| [180] | Recommendations for Prevention and Control of Influenza in Children, 2022–2023 [J]. Pediatrics, 2022, 150(4). | Irrelevant study |
| [181] | National Institute for Health and Care Research to fund new phase of platform trial to assess treatments for severe flu [J]. Pharmaceutical Journal, 2022, 309(7967). | Irrelevant study |
| [182] | Influenza vaccine for 2022-2023 [J]. The Medical letter on drugs and therapeutics, 2022, 64(1660): 153-157. | Irrelevant study |
| [183] | Zyryanovu S K, Butranova O I, Gaidai D S, Kryshen K L. Pharmacotherapy for acute respiratory infections caused by influenza viruses: current possibilities [J]. Terapevticheskii arkhiv, 2021, 93(1): 114-124. | Irrelevant study |
| [184] | Zaraket H, Hurt A C, Clinch B, Barr I, Lee N. Burden of influenza B virus infection and considerations for clinical management [J]. Antiviral research, 2021, 185: 104970. | Irrelevant study |
| [185] | Yuen E, Gudis D A, Rowan N R, Nguyen S A, Schlosser R J. Viral Infections of the Upper Airway in the Setting of COVID-19: A Primer for Rhinologists [J]. American Journal of Rhinology and Allergy, 2021, 35(1): 122-131. | Irrelevant study |
| [186] | Whitehead T, Goldwire M A. Influenza in the Age of COVID-19 [Z]. 2021: 31-35 | Irrelevant study |
| [187] | Terrier O, Slama-Schwok A. Anti-Influenza Drug Discovery and Development: Targeting the Virus and Its Host by All Possible Means [Z]. 2021: 195-218.10.1007/978-981-16-0267-2_8 | Irrelevant study |
| [188] | Tejada S, Tejo A M, Peña-López Y, Forero C G, Corbella X, Rello J. Neuraminidase inhibitors and single dose baloxavir are effective and safe in uncomplicated influenza: a meta-analysis of randomized controlled trials [J]. Expert Review of Clinical Pharmacology, 2021, 14(7): 901-918. | Irrelevant study |
| [189] | Tanaka H, Ohyama K, Horikomi Y, Ishii T. Association between anaphylaxis and anti-influenza drug use: An analysis of the Japanese Adverse Drug Event Report database [J]. Drug Discoveries and Therapeutics, 2021, 15(3): 150-155. | Irrelevant study |
| [190] | Takeuchi M, Kawakami K. Association of baloxavir marboxil prescription with subsequent medical resource utilization among school-aged children with influenza [J]. Pharmacoepidemiology and drug safety, 2021, 30(6): 779-786. | Irrelevant study |
| [191] | Takeuchi M, Kawakami K. Baloxavir marboxil vs neuraminidase inhibitors prescription on subsequent medical resource utilization among school-aged children with influenza: Analysis of two consecutive seasons in Japan [J]. Pharmacoepidemiology and drug safety, 2021, 30(SUPPL 1): 154. | Irrelevant study |
| [192] | Takashita E. Influenza Polymerase Inhibitors: Mechanisms of Action and Resistance [J]. COLD SPRING HARBOR PERSPECTIVES IN MEDICINE, 2021, 11(5). | Irrelevant study |
| [193] | Svyatchenko S V, Goncharova N I, Marchenko V Y, Kolosova N P, Shvalov A N, Kovrizhkina V L, et al. An influenza A(H5N8) virus isolated during an outbreak at a poultry farm in Russia in 2017 has an N294S substitution in the neuraminidase and shows reduced susceptibility to oseltamivir [J]. Antiviral research, 2021, 191: 105079. | Irrelevant study |
| [194] | Suzuki S, Nguyen C T, Ogata-Nakahara A, Shibata A, Osaka H, Ishigaki H, et al. Efficacy of a Cap-Dependent Endonuclease Inhibitor and Neuraminidase Inhibitors against H7N9 Highly Pathogenic Avian Influenza Virus Causing Severe Viral Pneumonia in Cynomolgus Macaques [J]. Antimicrobial agents and chemotherapy, 2021, 65(3). | Irrelevant study |
| [195] | Sunagawa S, Iha Y, Kinjo T, Nakamura K, Fujita J. Role of zanamivir is likely to end in Okinawa, Japan [J]. Respiratory investigation, 2021, 59(1): 159-161. |  |
| [196] | Sugawara T, Ohkusa Y, Taniguchi K, Miyazaki C, Kato Y, Okabe N. Association of bleeding symptoms during influenza infection and administered drugs [J]. Drug Discoveries and Therapeutics, 2021, 15(5): 261-267. | Irrelevant study |
| [197] | Soh Y Q S, Malone K D, Eguia R T, Bloom J D. Comprehensive Profiling of Mutations to Influenza Virus PB2 That Confer Resistance to the Cap-Binding Inhibitor Pimodivir [J]. VIRUSES-BASEL, 2021, 13(7). | Irrelevant study |
| [198] | Skrzeczek A, Ikeoka H, Hirotsu N, Ansaripour A, Aballea S, Onishi Y, et al. Cost-effectiveness of baloxavir marboxil compared to laninamivir for the treatment of influenza in Japan [J]. JOURNAL OF INFECTION AND CHEMOTHERAPY, 2021, 27(2): 296-305. | Wrong outcome |
| [199] | Shirata M, Ito I, Hirai T. Assessment of Anti-Influenza Drug Prescriptions for Postexposure Prophylaxis Against Household Transmission of Influenza Virus [J]. Clinical Infectious Diseases, 2021, 73(7): E1766. | Wrong study design |
| [200] | Shiomi T, Fujiwara M, Kitanishi Y, Miyazawa S, Komeda T, Miyauchi H, et al. Hospital revisits and additional prescriptions in outpatients with influenza after treatment with anti-influenza drugs: Japanese health insurance claims database [J]. Japanese Journal of Chemotherapy, 2021, 69(4): 318-328. | Wrong study design |
| [201] | Rong L. Development of 4-(aroylamino)piperidine-based entry inhibitors as anti-influenza therapeutics [J]. 2021. | Irrelevant study |
| [202] | Portsmouth S, Hayden F G, Kawaguchi K, Ishibashi T, Kinoshita M, Shishido T, et al. Baloxavir Treatment in Adolescents With Acute Influenza: Subgroup Analysis From the CAPSTONE-1 Trial [J]. Journal of the Pediatric Infectious Diseases Society, 2021, 10(4): 477-484. | Wrong study design |
| [203] | Pascua P N Q, Jones J C, Marathe B M, Seiler P, Caufield W V, Freeman B B, III, et al. Baloxavir Treatment Delays Influenza B Virus Transmission in Ferrets and Results in Limited Generation of Drug-Resistant Variants [J]. Antimicrobial agents and chemotherapy, 2021, 65(11). | Wrong study design |
| [204] | Park J H, Kim B, Antigua K J C, Jeong J H, Kim C I, Choi W S, et al. Baloxavir-oseltamivir combination therapy inhibits the emergence of resistant substitutions in influenza A virus PA gene in a mouse model [J]. Antiviral research, 2021, 193: 105126. | Wrong study design |
| [205] | Palomba E, Castelli V, Renisi G, Bandera A, Lombardi A, Gori A. Antiviral Treatments for Influenza [J]. Seminars in Respiratory and Critical Care Medicine, 2021, 42(6): 859-872. | Irrelevant study |
| [206] | Osada H, Chon I, Phyu W W, Wagatsuma K, Nagata N, Kawashima T, et al. Development of cycling probe based real-time PCR methodology for influenza A viruses possessing the PA/I38T amino acid substitution associated with reduced baloxavir susceptibility [J]. Antiviral research, 2021, 188. | Irrelevant study |
| [207] | O'Driscoll L S, Martin-Loeches I. Management of Severe Influenza [J]. Seminars in Respiratory and Critical Care Medicine, 2021, 42(6): 771-787. | Irrelevant study |
| [208] | Mehmood I, Ijaz M, Ahmad S, Ahmed T, Bari A, Abro A, et al. Sars-cov-2: An update on genomics, risk assessment, potential therapeutics and vaccine development [J]. International journal of environmental research and public health, 2021, 18(4): 1-24. |  |
| [209] | Masterson C H, Ceccato A, Artigas A, dos Santos C, Rocco P R, Enes S R, et al. Mesenchymal stem/stromal cell-based therapies for severe viral pneumonia: therapeutic potential and challenges [J]. INTENSIVE CARE MEDICINE EXPERIMENTAL, 2021, 9(1). | Irrelevant study |
| [210] | Marinelli T M, Kumar D. Influenza Virus Infection and Transplantation [J]. Transplantation, 2021, 105(5): 968-978. | Irrelevant study |
| [211] | Macesic N, Laplante J M, Aaron J G, DiMango E A, Miko B A, Pereira M R, et al. Baloxavir treatment of oseltamivir-resistant influenza A/H1pdm09 in two immunocompromised patients [J]. Transplant Infectious Disease, 2021, 23(3). | Wrong study design |
| [212] | Liu J-W, Lin S-H, Wang L-C, Chiu H-Y, Lee J-A. Comparison of Antiviral Agents for Seasonal Influenza Outcomes in Healthy Adults and Children A Systematic Review and Network Meta-analysis [J]. JAMA network open, 2021, 4(8). | Wrong study design |
| [213] | Liu J W, Lin S H, Wang L C, Chiu H Y, Lee J A. Comparison of Antiviral Agents for Seasonal Influenza Outcomes in Healthy Adults and Children: A Systematic Review and Network Meta-analysis [J]. JAMA network open, 2021, 4(8): e2119151. | Wrong study design |
| [214] | Lee L Y Y, Zhou J, Koszalka P, Frise R, Farrukee R, Baba K, et al. Evaluating the fitness of PA/I38T-substituted influenza A viruses with reduced baloxavir susceptibility in a competitive mixtures ferret model [J]. PLoS pathogens, 2021, 17(5). | Wrong study design |
| [215] | Komeda T, Takazono T, Hosogaya N, Ogura E, Fujiwara M, Miyauchi H, et al. Comparison of Household Transmission of Influenza Virus From Index Patients Treated With Baloxavir Marboxil or Neuraminidase Inhibitors: A Health Insurance Claims Database Study [J]. Clinical infectious diseases : an official publication of the Infectious Diseases Society of America, 2021, 72(11): e859-e867. | Wrong study design |
| [216] | Komeda T, Takazono T, Hosogaya N, Miyazaki T, Ogura E, Iwata S, et al. Comparison of Hospitalization Incidence in Influenza Outpatients Treated with Baloxavir Marboxil or Neuraminidase Inhibitors: A Health Insurance Claims Database Study [J]. Clinical Infectious Diseases, 2021, 73(5): E1181-E1190. | Wrong study design |
| [217] | Ivashchenko A A, Mitkin O D, Jones J C, Nikitin A V, Koryakova A G, Karapetian R N, et al. Synthesis, inhibitory activity and oral dosing formulation of AV5124, the structural analogue of influenza virus endonuclease inhibitor baloxavir [J]. The Journal of antimicrobial chemotherapy, 2021, 76(4): 1010-1018. | Wrong study design |
| [218] | Ison M G, Hayden F G, Hay A J, Gubareva L V, Govorkova E A, Takashita E, et al. Influenza polymerase inhibitor resistance: Assessment of the current state of the art - A report of the isirv Antiviral group [J]. Antiviral research, 2021, 194: 105158. | Irrelevant study |
| [219] | Ison M G, de Cassia Castro R, Hurt A C, Ko J, Yau L, Kuhlbusch K, et al. Outcomes of Influenza with Treatment in Patients with Diabetes: Subgroup Analysis of the Phase 3 CAPSTONE-2 Trial [J]. Metabolism: clinical and experimental, 2021, 116. | Irrelevant study |
| [220] | Ishiguro N, Morioka I, Nakano T, Furukawa M, Tanaka S, Kinoshita M, et al. Clinical and virological outcomes with baloxavir compared with oseltamivir in pediatric patients aged 6 to &lt; 12 years with influenza: an open-label, randomized, active-controlled trial protocol [J]. BMC INFECTIOUS DISEASES, 2021, 21(1). | Full text not obtainable |
| [221] | Hosogaya N, Takazono T, Yokomasu A, Hiroi S, Ikeoka H, Iwasaki K, et al. Estimation of the value of convenience in taking influenza antivirals in Japanese adult patients between baloxavir marboxil and neuraminidase inhibitors using a conjoint analysis [J]. Journal of Medical Economics, 2021, 24(1): 244-254. | Wrong population |
| [222] | Holmes E C, Hurt A C, Dobbie Z, Clinch B, Oxford J S, Piedra P A. Understanding the Impact of Resistance to Influenza Antivirals [J]. Clinical microbiology reviews, 2021, 34(2). | Irrelevant study |
| [223] | Heimonen J, McCulloch D J, O'Hanlon J, Kim A E, Emanuels A, Wilcox N, et al. A remote household-based approach to influenza self-testing and antiviral treatment [J]. Influenza and other respiratory viruses, 2021, 15(4): 469-477. | Irrelevant study |
| [224] | Hara A, Hara K, Komeda T, Ogura E, Miyazawa S, Kobayashi C, et al. Comparison of the incidence of bleeding between anti-influenza drugs among outpatients for the influenza virus infection: A retrospective study using an employment-based health insurance claims database in Japan [J]. Pharmacoepidemiology and drug safety, 2021, 30(SUPPL 1): 182-183. | Irrelevant study |
| [225] | Fujita M, Matsumoto H, Inafuku Y, Toyama J, Fujita J. A retrospective observational study of the treatment of a nosocomial infection caused by oseltamivir-resistant influenza virus A with baloxavir marboxil (vol 58, pg 403, 2020) [J]. Respiratory investigation, 2021, 59(5): 708-708. | Wrong study design |
| [226] | El Feghaly R E, Nolen J D, Lee B R, Abraham G, Nedved A, Hassan F, et al. Impact of Rapid Influenza Molecular Testing on Management in Pediatric Acute Care Settings [J]. Journal of Pediatrics, 2021, 228: 271-277.e271. | Irrelevant study |
| [227] | Maurice A, Martin-Blais R, Halasa N. Preparing for the 2020—2021 influenza season [J]. Pediatric Transplantation, 2021, 25(5). | Irrelevant study |
| [228] | Comm Infect D. Recommendations for Prevention and Control of Influenza in Children, 2021-2022 [J]. Pediatrics, 2021, 148(4). | Irrelevant study |
| [229] | Clementi N, Ghosh S, De Santis M, Castelli M, Criscuolo E, Zanoni I, et al. Viral respiratory pathogens and lung injury [J]. Clinical microbiology reviews, 2021, 34(3). | Irrelevant study |
| [230] | Chua K H, Mohamed I N, Mohd Yunus M H, Shafinaz Md Nor N, Kamil K, Ugusman A, et al. The Anti-Viral and Anti-Inflammatory Properties of Edible Bird’s Nest in Influenza and Coronavirus Infections: From Pre-Clinical to Potential Clinical Application [J]. Frontiers in pharmacology, 2021, 12. | Irrelevant study |
| [231] | Chtita S, Belhassan A, Aouidate A, Belaidi S, Bouachrine M, Lakhlifi T. Discovery of potent sars-cov-2 inhibitors from approved antiviral drugs via docking and virtual screening [J]. Combinatorial Chemistry and High Throughput Screening, 2021, 24(3): 441-454. | Irrelevant study |
| [232] | Chow E J, Beigi R H, Riley L E, Uyeki T M. Clinical Effectiveness and Safety of Antivirals for Influenza in Pregnancy [J]. Open Forum Infectious Diseases, 2021, 8(6). | Irrelevant study |
| [233] | Chotpitayasunondh T, Fischer T K, Heraud J-M, Hurt A C, Monto A S, Osterhaus A, et al. Influenza and COVID-19: What does co-existence mean? [J]. Influenza and other respiratory viruses, 2021, 15(3): 407-412. | Irrelevant study |
| [234] | Chong Y, Kawai N, Tani N, Bando T, Takasaki Y, Shindo S, et al. Virological and clinical outcomes in outpatients treated with baloxavir or oseltamivir: A Japanese multicenter study in the 2019–2020 influenza season [J]. Antiviral research, 2021, 192. | Wrong population |
| [235] | Center M D A C. Baloxavir and Oseltamivir for the Treatment of Severe Influenza Infection in Immunocompromised Patients [J]. clinicaltrialsgov, 2021. | Wrong population |
| [236] | Caceres C J, Hu Y, Cardenas-Garcia S, Wu X, Tan H, Carnaccini S, et al. Rational design of a deuterium-containing M2-S31N channel blocker UAWJ280 with <i>in vivo</i> antiviral efficacy against both oseltamivir sensitive and -resistant influenza A viruses [J]. Emerging microbes & infections, 2021, 10(1): 1832-1848. | Wrong population |
| [237] | Bulloch M, Hartley C, Elston R. Evaluation of clinical trials including geriatrics for treatment and prophylaxis of influenza [J]. Critical Care Medicine, 2021, 49(1 SUPPL 1): 332. | Irrelevant study |
| [238] | Beigel J H, Hayden F G. Influenza Therapeutics in Clinical Practice-Challenges and Recent Advances [J]. COLD SPRING HARBOR PERSPECTIVES IN MEDICINE, 2021, 11(4). | Irrelevant study |
| [239] | Apaydın Ç B, Tansuyu M, Cesur Z, Naesens L, Göktaş F. Design, synthesis and anti-influenza virus activity of furan-substituted spirothiazolidinones [J]. Bioorganic chemistry, 2021, 112. | Irrelevant study |
| [240] | Apaydin C B, Cinar G, Cihan-Ustundag G. Small-molecule Antiviral Agents in Ongoing Clinical Trials for COVID-19 [J]. Current drug targets, 2021, 22(17): 1986-2005. | Irrelevant study |
| [241] | Ando Y, Noshi T, Sato K, Ishibashi T, Yoshida Y, Hasegawa T, et al. Pharmacokinetic and pharmacodynamic analysis of baloxavir marboxil, a novel cap-dependent endonuclease inhibitor, in a murine model of influenza virus infection [J]. The Journal of antimicrobial chemotherapy, 2021, 76(1): 189-198. | Wrong comparator |
| [242] | Amin Hussen N H. Docking study of naringin binding with COVID-19 main protease enzyme [J]. Iraqi Journal of Pharmaceutical Sciences, 2021, 29(2): 231-238. | Irrelevant study |
| [243] | Aliberti S, Dela Cruz C S, Amati F, Sotgiu G, Restrepo M I. Community-acquired pneumonia [J]. The Lancet, 2021, 398(10303): 906-919. | Irrelevant study |
| [244] | Adams S Y, Davis T W, Lechner B E. Perspectives on Race and Medicine in the NICU [J]. Pediatrics, 2021, 147(3). | Irrelevant study |
| [245] | Baloxavir (Xofluza) for post-exposure prophylaxis of influenza [J]. Medical Letter on Drugs and Therapeutics, 2021, 63(1615): 2-3. | Wrong research type |
| [246] | Erratum regarding previously published articles (Respiratory Investigation (2020) 58(3) (204–211), (S2212534520300071), (10.1016/j.resinv.2020.01.002)) [J]. Respiratory investigation, 2021, 59(5): 708. | Irrelevant study |
| [247] | Influenza vaccine for 2021-2022 [J]. The Medical letter on drugs and therapeutics, 2021, 63(1634): 153-157. | Irrelevant study |
| [248] | Zhao L, Yan Y, Dai Q, Li X, Xu K, Zou G, et al. Development of Novel Anti-influenza Thiazolides with Relatively Broad-Spectrum Antiviral Potentials [J]. Antimicrobial agents and chemotherapy, 2020, 64(7). | Irrelevant study |
| [249] | Zhao L, Che J, Zhang Q, Li Y, Guo X, Chen L, et al. Identification of Novel Influenza Polymerase PB2 Inhibitors Using a Cascade Docking Virtual Screening Approach [J]. Molecules (Basel, Switzerland), 2020, 25(22). | Irrelevant study |
| [250] | Yoshino Y, Kitazawa T, Ota Y. Clinical efficacy of baloxavir marboxil in the treatment of seasonal influenza in adult patients: A prospective observational study [J]. International Journal of General Medicine, 2020, 13: 735-741. | Wrong population |
| [251] | Yoshimura Y, Sasaki H, Horiuchi H, Miyata N, Kawakami C, Usuku S, et al. Early combination treatment with baloxavir and peramivir for hospitalized adults with influenza A in Yokohama, Japan [J]. European Journal of Clinical Microbiology and Infectious Diseases, 2020, 39(9): 1637-1640. | Wrong population |
| [252] | Yoshii N, Tochino Y, Fujioka M, Sakazaki H, Maruyama N, Asai K, et al. The comparison of the efficacy of baloxavir and neuraminidase inhibitors for patients with influenza a in clinical practice [J]. Internal Medicine, 2020, 59(12): 1509-1513. | Wrong population |
| [253] | Yokoyama T, Sakaguchi H, Ishibashi T, Shishido T, Piedra P A, Sato C, et al. Baloxavir Marboxil 2% Granules in Japanese Children With Influenza An Open-label Phase 3 Study [J]. PEDIATRIC INFECTIOUS DISEASE JOURNAL, 2020, 39(8): 706-712. | Wrong intervention |
| [254] | Yano T, Ochiai H, Akachi S, Matsumura Y. Polymerase acidic subunit i38t mutant influenza a(H3N2) virus isolated from a pediatric patient without prior baloxavir marboxil treatment in mie prefecture (November 2018) [J]. Japanese journal of infectious diseases, 2020, 73(5): 383-385. | Wrong study design |
| [255] | Yang Lee L Y, Zhou J, Frise R, Goldhill D H, Koszalka P, Mifsud E J, et al. Baloxavir treatment of ferrets infected with influenza A(H1N1)pdm09 virus reduces onward transmission [J]. PLoS pathogens, 2020, 16(4). | Wrong study design |
| [256] | Van Poelvoorde L A E, Saelens X, Thomas I, Roosens N H. Next-Generation Sequencing: An Eye-Opener for the Surveillance of Antiviral Resistance in Influenza [J]. Trends in Biotechnology, 2020, 38(4): 360-367. | Irrelevant study |
| [257] | Umemura T, Mutoh Y, Kawamura T, Saito M, Tsuboi T, Kozaki K, et al. The effect of baloxavir marboxil on household transmission with influenza infection [J]. Journal of Infection and Public Health, 2020, 13(2): 351. | Wrong outcome |
| [258] | Umemura T, Mutoh Y, Kawamura T, Saito M, Mizuno T, Ota A, et al. Efficacy of baloxavir marboxil on household transmission of influenza infection [J]. Journal of pharmaceutical health care and sciences, 2020, 6: 21. | Wrong outcome |
| [259] | Uehara T, Hayden F G, Kawaguchi K, Omoto S, Hurt A C, De Jong M D, et al. Treatment-Emergent Influenza Variant Viruses With Reduced Baloxavir Susceptibility: Impact on Clinical and Virologic Outcomes in Uncomplicated Influenza [J]. The Journal of infectious diseases, 2020, 221(3): 346-355. | Wrong study design |
| [260] | Twabela A, Okamatsu M, Matsuno K, Isoda N, Sakoda Y. Evaluation of Baloxavir Marboxil and Peramivir for the Treatment of High Pathogenicity Avian Influenza in Chickens [J]. VIRUSES-BASEL, 2020, 12(12). | Wrong study design |
| [261] | Toots M, Plemper R K. Next-generation direct-acting influenza therapeutics [J]. TRANSLATIONAL RESEARCH, 2020, 220: 33-42. | Irrelevant study |
| [262] | Tobar Vega P, Caldeira E, Abad H, Saad P, Lachance E. Oseltamivir and baloxavir: Dual treatment for rapidly developing ARDS on a patient with renal disease [J]. IDCases, 2020, 21. | Irrelevant study |
| [263] | Tillotson G S. A new dawn for the management of influenza? [J]. The Lancet Infectious Diseases, 2020, 20(10): 1112-1114. | Irrelevant study |
| [264] | Teoh S L, Lim Y H, Lai N M, Lee S W H. Directly Acting Antivirals for COVID-19: Where Do We Stand? [J]. Frontiers in Microbiology, 2020, 11. | Irrelevant study |
| [265] | Tenforde M W, Cummings C N, Sutton M, Kim S, Maslar A, Alden N B, et al. Influenza antiviral use in patients hospitalized with laboratory-confirmed influenza in the United States, flusurv-net, 2015-2019 [J]. Open Forum Infectious Diseases, 2020, 7(SUPPL 1): S759. | Irrelevant study |
| [266] | Takashita E, Fujisaki S, Yokoyama M, Shirakura M, Morita H, Nakamura K, et al. In vitro characterization of multidrug-resistant influenza a(H1n1)pdm09 viruses carrying a dual neuraminidase mutation isolated from immunocompromised patients [J]. Pathogens (Basel, Switzerland), 2020, 9(9): 1-13. | Irrelevant study |
| [267] | Takashita E, Daniels R S, Fujisaki S, Gregory V, Gubareva L V, Huang W, et al. Global update on the susceptibilities of human influenza viruses to neuraminidase inhibitors and the cap-dependent endonuclease inhibitor baloxavir, 2017-2018 [J]. Antiviral research, 2020, 175: 104718. | Irrelevant study |
| [268] | Szollosi D, Bill A. Potential Role of Endonuclease Inhibition and Other Targets in the Treatment of Influenza [J]. Current drug targets, 2020, 21(2): 202-211. | Irrelevant study |
| [269] | Spieler E E, Moritz E, Stertz S, Hale B G. Application of a Biologically Contained Reporter System To Study Gain-of-Function H5N1 Influenza A Viruses with Pandemic Potential [J]. mSphere, 2020, 5(4). | Irrelevant study |
| [270] | Shamim S, Khan M, Kharaba Z J, Ijaz M, Murtaza G. Potential strategies for combating COVID-19 [J]. Archives of virology, 2020, 165(11): 2419-2438. | Irrelevant study |
| [271] | Shah S, McManus D, Bejou N, Tirmizi S, Rouse G E, Lemieux S M, et al. Clinical outcomes of baloxavir versus oseltamivir in patients hospitalized with influenza A [J]. Journal of Antimicrobial Chemotherapy, 2020, 75(10): 3015-3022. | Wrong population |
| [272] | Shah S S, Raddawi M A R Y. A DIAGNOSTIC JOURNEY FOR PROGRESSIVE HYPOXEMIC RESPIRATORY FAILURE IN PATIENT WITH CYSTIC FIBROSIS AND LUNG TRANSPLANT [Z]. 2024: A1987-A1988.10.1016/j.chest.2024.06.1236 | Irrelevant study |
| [273] | Salvatore M, Laplante J M, Soave R, Orfali N, Plate M, van Besien K, et al. Baloxavir for the treatment of Influenza in allogeneic hematopoietic stem cell transplant recipients previously treated with oseltamivir [J]. Transplant infectious disease : an official journal of the Transplantation Society, 2020, 22(4): e13336. | Wrong population |
| [274] | Salvatore M, Laplante J M, Soave R, Orfali N, Plate M, Besien K, et al. Baloxavir for the treatment of Influenza in allogeneic hematopoietic stem cell transplant recipients previously treated with oseltamivir [J]. TRANSPLANT INFECTIOUS DISEASE, 2020, 22(4). | Wrong population |
| [275] | Sato M. Pharmacologic background and clinical issue of anti-influenza drugs [J]. Fukushima journal of medical science, 2024. | Irrelevant study |
| [276] | Park J-G, Ye C, Piepenbrink M S, Nogales A, Wang H, Shuen M, et al. A Broad and Potent H1-Specific Human Monoclonal Antibody Produced in Plants Prevents Influenza Virus Infection and Transmission in Guinea Pigs [J]. VIRUSES-BASEL, 2020, 12(2). | Irrelevant study |
| [277] | Papenburg J, Mubareka S, Allen U D, Skowronski D M, Stiver H G, Aoki F Y, et al. Guidance on the use of antiviral agents for the 2019–2020 influenza season [J]. JAMMI, 2020, 5(2): 57-60. | Irrelevant study |
| [278] | O'Sullivan S, Torres A, Rodriguez A, Martin-Loeches I. Influenza management with new therapies [J]. Current opinion in pulmonary medicine, 2020, 26(3): 215-221. | Irrelevant study |
| [279] | Omrani M, Keshavarz M, Nejad Ebrahimi S, Mehrabi M, McGaw L J, Ali Abdalla M, et al. Potential Natural Products Against Respiratory Viruses: A Perspective to Develop Anti-COVID-19 Medicines [J]. Frontiers in pharmacology, 2020, 11. | Irrelevant study |
| [280] | Odnovorov A, Garaev T, Grebennikova T, Pleteneva T. Prospects for specific influenza treatment [J]. Systematic Reviews in Pharmacy, 2020, 11(2): 242-248. | Irrelevant study |
| [281] | Norikoshi Y, Ikeda T, Sasahara K, Hamada M, Torigoe E, Nagae M, et al. A Comparison of the Frequency of Prescription and Pharmacy Revisits between Baloxavir Marboxil and Neuraminidase Inhibitors in Influenza-Infected Pediatric Patients during the 2019-2020 Influenza Season [J]. Biological & pharmaceutical bulletin, 2020, 43(12): 1960-1965. | Wrong outcome |
| [282] | Newman K L, Rogers J H, McCulloch D, Wilcox N, Englund J A, Boeckh M, et al. Point-of-care molecular testing and antiviral treatment of influenza in residents of homeless shelters in Seattle, WA: study protocol for a stepped-wedge cluster-randomized controlled trial [J]. Trials, 2020, 21(1). | Irrelevant study |
| [283] | Neuberger E, Wallick C, Chawla D, De Cassia Castro R. Real-world comparative effectiveness of baloxavir marboxil versus oseltamivir on influenza-related complication and resource utilization [J]. Open Forum Infectious Diseases, 2020, 7(SUPPL 1): S761-S762. | Wrong outcome |
| [284] | Nct. Randomised Evaluation of COVID-19 Therapy [J]. https://clinicaltrialsgov/ct2/show/NCT04381936, 2020. | Irrelevant study |
| [285] | Mubareka S, Aoki F Y, Allen U D, Hatchette T F, Papenburg J, Evans G A. 2020–2021 ammi canada guidance on the use of antiviral drugs for influenza in the setting of co-circulation of seasonal influenza and sars-cov-2 viruses in canada [J]. JAMMI, 2020, 5(4): 214-222. | Irrelevant study |
| [286] | Mhamdi Z, Fausther-Bovendo H, Uyar O, Carbonneau J, Venable M-C, Abed Y, et al. Effects of Different Drug Combinations in Immunodeficient Mice Infected with an Influenza A/H3N2 Virus [J]. Microorganisms, 2020, 8(12). | Irrelevant study |
| [287] | Levin J, Amundson K, Borchardt A, Lam T, Brady T, Abelovski E, et al. Evaluation of CD377, a Novel Antiviral Fc-Conjugate (AVC), In Vitro Activity and In Vivo Efficacy in Immune-Competent and -Deficient (SCID) Lethal Mouse Models [J]. Open Forum Infectious Diseases, 2020, 7(SUPPL 1): S654. | Irrelevant study |
| [288] | Lee L Y Y, Zhou J, Frise R, Goldhill D H, Koszalka P, Mifsud E J, et al. Baloxavir treatment of ferrets infected with influenza A(H1N1)pdm09 virus reduces onward transmission [J]. PLoS pathogens, 2020, 16(4): e1008395. | Wrong study design |
| [289] | Koshimichi H, Retout S, Cosson V, Duval V, De Buck S, Tsuda Y, et al. Population pharmacokinetics and exposure-response relationships of baloxavir marboxil in influenza patients at high risk of complications [J]. Antimicrobial agents and chemotherapy, 2020, 64(7). | Wrong population |
| [290] | Korsun N, Daniels R, Angelova S, Ermetal B, Grigorova I, Voleva S, et al. Genetic diversity of influenza A viruses circulating in Bulgaria during the 2018–2019 winter season [J]. Journal of Medical Microbiology, 2020, 69(7): 986-998. | Wrong outcome |
| [291] | Kormuth K A, Lakdawala S S. Emerging antiviral resistance [J]. Nature Microbiology, 2020, 5(1): 4-5. | Irrelevant study |
| [292] | Kitano M, Matsuzaki T, Oka R, Baba K, Noda T, Yoshida Y, et al. The antiviral effects of baloxavir marboxil against influenza A virus infection in ferrets [J]. Influenza and other respiratory viruses, 2020, 14(6): 710-719. | Wrong study design |
| [293] | Kamioka Y, Kashiwagura S, Seki M. Reduced Prescription of Baloxavir After Suspected Prevalence of a Baloxavir-Resistant Influenza Virus Strain and the Emergence of SARS-CoV-2 in a Tertiary Hospital in Japan [J]. Clinical pharmacology : advances and applications, 2020, 12: 131-134. | Wrong study design |
| [294] | jRcts. A randomized, controlled study of baloxavir marboxil compared with oseltamivir in patients with influenza virus infection aged 75 years and older [J]. https://trialsearchwhoint/Trial2aspx?TrialID=JPRN-jRCTs071200034, 2020. | Wrong population |
| [295] | Sarker J, Carkovic E, Ptaszek K, Lee T A. Antiviral influenza treatments and hemorrhage-related adverse events in the United States Food and Drug Administration Adverse Event Reporting System (FAERS) database [J]. Pharmacotherapy, 2024, 44(5): 383-393. | Wrong study design |
| [296] | Ikematsu H, Kawai N, Tani N, Chong Y, Iwaki N, Bando T, et al. Duration of fever and PA/I38X-substituted virus emergence in patients treated with baloxavir in the 2018–2019 influenza season [J]. Journal of Infection and Chemotherapy, 2020, 26(4): 400-402. | Wrong study design |
| [297] | Ikematsu H, Hayden F G, Kawaguchi K, Kinoshita M, de Jong M D, Lee N, et al. Baloxavir marboxil for prophylaxis against influenza in household contacts [J]. New England Journal of Medicine, 2020, 383(4): 309-320. | Wrong outcome |
| [298] | Hirotsu N, Sakaguchi H, Sato C, Ishibashi T, Baba K, Omoto S, et al. Baloxavir Marboxil in Japanese Pediatric Patients With Influenza: Safety and Clinical and Virologic Outcomes [J]. CLINICAL INFECTIOUS DISEASES, 2020, 71(4): 971-981. | Wrong population |
| [299] | Harada N, Shibata W, Koh H, Takashita E, Fujisaki S, Okamura H, et al. Successful treatment with baloxavir marboxil of a patient with peramivir-resistant influenza A/H3N2 with a dual E119D/R292K substitution after allogeneic hematopoietic cell transplantation: a case report [J]. BMC Infect Dis, 2020, 20(1): 478. | Wrong research type |
| [300] | Gubareva L V, Fry A M. Baloxavir and Treatment-Emergent Resistance: Public Health Insights and Next Steps [J]. JOURNAL OF INFECTIOUS DISEASES, 2020, 221(3): 337-339. | Wrong research type |
| [301] | Fujita M, Matsumoto H, Inafuku Y, Toyama J, Fujita J. A retrospective observational study of the treatment of a nosocomial infection caused by oseltamivir-resistant influenza virus A with baloxavir marboxil [J]. Respiratory investigation, 2020, 58(5): 403-408. | Wrong outcome |
| [302] | Fujita J. Introducing the new anti-influenza drug, baloxavir marboxil [J]. Respiratory investigation, 2020, 58(1): 1-3. | Wrong research type |
| [303] | Fujita J. Clinical application of baloxavir marboxil in the treatment of influenza [J]. Respiratory investigation, 2020, 58(5): 301-304. | Wrong research type |
| [304] | Fang Q, Wang D. Advanced researches on the inhibition of influenza virus by Favipiravir and Baloxavir [J]. BIOSAFETY AND HEALTH, 2020, 2(2): 64-70. | Wrong comparator |
| [305] | Ehrlich H, Boneva D, Elkbuli A. The intersection of viral illnesses: A seasonal influenza epidemic amidst the COVID-19 pandemic [J]. Annals of Medicine and Surgery, 2020, 60: 41-43. | Irrelevant study |
| [306] | Dürrwald R, Wedde M, Biere B, Oh D Y, Heßler-Klee M, Geidel C, et al. Zoonotic infection with swine A/H1avN1 influenza virus in a child, Germany, June 2020 [J]. Eurosurveillance, 2020, 25(42). | Irrelevant study |
| [307] | Du Z, Meyers L A, Nugent C, Meyers L A, Galvani A P, Krug R M, et al. Modeling mitigation of influenza epidemics by baloxavir [J]. Nature Communications, 2020, 11(1): 2750. | Wrong study design |
| [308] | Maurice A, Halasa N. Preparing for the 2019-2020 influenza season [J]. Pediatric Transplantation, 2020, 24(1). | Irrelevant study |
| [309] | Clausen E S, Zaffiri L. Infection prophylaxis and management of viral infection [J]. Annals of translational medicine, 2020, 8(6). | Irrelevant study |
| [310] | Checkmahomed L, Padey B, Pizzorno A, Terrier O, Rosa-Calatrava M, Abed Y, et al. In Vitro Combinations of Baloxavir Acid and Other Inhibitors against Seasonal Influenza A Viruses [J]. Viruses, 2020, 12(10). | Wrong study design |
| [311] | Checkmahomed L, M'Hamdi Z, Carbonneau J, Venable M C, Baz M, Abed Y, et al. Impact of the Baloxavir-Resistant Polymerase Acid I38T Substitution on the Fitness of Contemporary Influenza A(H1N1)pdm09 and A(H3N2) Strains [J]. The Journal of infectious diseases, 2020, 221(1): 63-70. | Wrong study design |
| [312] | Bhadru B, Rao V V, Vidyadhara S. DEVELOPMENT AND VALIDATION FOR HIGH-PERFORMANCE MASS SPECTROMETRY METHOD FOR DETERMINATION OF BALOXAVIR MARBOXIL IN BIOLOGICAL MATRICES [J]. INTERNATIONAL JOURNAL OF PHARMACEUTICAL SCIENCES AND RESEARCH, 2020, 11(5): 2324-2331. | Irrelevant study |
| [313] | Jiang Tingting, Zhang Ni, Su Hui, Li Yanping, Liu Yao. Mining and Analysis of Adverse Event Signals of Oseltamivir, Zanamivir and Baloxavir Marboxil [J]. 2024, 35, China Pharmacy. | Wrong study type |
| [314] | Baba K, Oka R, Shano S, Omoto S, Noshi T, Shishido T, et al. Carryover effects of baloxavir acid in human nasopharyngeal/pharyngeal swabs on infectious titer testing of influenza virus [J]. Influenza and other respiratory viruses, 2020, 14(3): 353-357. | Irrelevant study |
| [315] | Ando S. Estimation of the effectiveness of quadrivalent influenza vaccines by distinguishing between influenza a (H1N1) pdm09 and Influenza A (H3N2) using rapid influenza diagnostic tests during the 2018-2019 season [J]. Internal Medicine, 2020, 59(7): 933-940. | Irrelevant study |
| [316] | Abraham G M, Morton J B, Saravolatz L D. Baloxavir: A novel antiviral agent in the treatment of influenza [J]. Clinical Infectious Diseases, 2020, 71(7): 1790-1794. | Wrong research type |
| [317] | open-label, active-controlled study of baloxavir in pediatric patients with influenza [J]. Japan Primary Registry Network, 2020. | Wrong research type |
| [318] | Antiviral drugs for influenza for 2020-2021 [J]. Medical Letter on Drugs and Therapeutics, 2020, 62(1610): 169-173. | Irrelevant study |
| [319] | Influenza vaccine for 2020-2021 [J]. The Medical letter on drugs and therapeutics, 2020, 62(1607): 145-150. | Irrelevant study |
| [320] | Antiviral drugs for influenza [J]. The Medical letter on drugs and therapeutics, 2020, 62(1589): 1-4. | Irrelevant study |
| [321 | Yoshino Y, Misu K, Wakabayashi Y, Ota Y, Kitazawa T. Evaluation of clinical course and heath-related quality-of-life following treatment with oseltamivir, laninamivir, and baloxavir marboxil in adult patients with seasonal influenza: Prospective observational study [J]. Open Forum Infectious Diseases, 2019, 6: S925. | Wrong population |
| [322] | Yang T. Baloxavir Marboxil: The First Cap-Dependent Endonuclease Inhibitor for the Treatment of Influenza [J]. Annals of Pharmacotherapy, 2019, 53(7): 754-759. | Wrong research type |
| [323] | Tauran Y, Ceron-Carrasco J P, Rhimi M, Perret F, Kim B, Collard D, et al. Size and Flexibility Define the Inhibition of the H3N2 Influenza Endonuclease Enzyme by Calix[n]arenes [J]. ANTIBIOTICS-BASEL, 2019, 8(2). | Irrelevant study |
| [324] | Taniguchi K, Ando Y, Nobori H, Toba S, Noshi T, Kobayashi M, et al. Inhibition of avian-origin influenza A(H7N9) virus by the novel cap-dependent endonuclease inhibitor baloxavir marboxil [J]. Scientific Reports, 2019, 9. | Wrong research type |
| [325] | Takashita E, Kawakami C, Ogawa R, Morita H, Fujisaki S, Shirakura M, et al. Influenza a(H3N2) virus exhibiting reduced susceptibility to baloxavir due to a polymerase acidic subunit I38T substitution detected from a hospitalized child without prior baloxavir treatment, Japan, January 2019 [J]. Eurosurveillance, 2019, 24(12). | Irrelevant study |
| [326] | Takashita E, Kawakami C, Morita H, Ogawa R, Fujisaki S, Shirakura M, et al. Detection of influenza A(H3N2) viruses exhibiting reduced susceptibility to the novel cap-dependent endonuclease inhibitor baloxavir in Japan, December 2018 [J]. Eurosurveillance, 2019, 24(3). | Irrelevant study |
| [327] | Takashita E, Ichikawa M, Morita H, Ogawa R, Fujisaki S, Shirakura M, et al. Human-to-human transmission of influenza A(H3N2) virus with reduced susceptibility to baloxavir, Japan, February 2019 [J]. Emerging infectious diseases, 2019, 25(11): 2108-2111. | Irrelevant study |
| [328] | Sucher A, Do A, Negash M. Latest clinical practice guidelines for seasonal influenza [Z]. 2019: 26-29 | Irrelevant study |
| [329] | Shah S, McManus D, Bejou N, Tirmizi S, Rouse G, Lemieux S, et al. Clinical outcomes of oseltamivir vs. Baloxavir in patients hospitalized with influenza A [J]. Open Forum Infectious Diseases, 2019, 6: S925. | Wrong population |
| [330] | Reina J, Reina N. [Baloxavir marboxil: a potent cap-dependent endonuclease inhibitor of influenza viruses] [J]. Revista espanola de quimioterapia : publicacion oficial de la Sociedad Espanola de Quimioterapia, 2019, 32(1): 1-5. | Wrong research type |
| [331] | Principi N, Camilloni B, Alunno A, Polinori I, Argentiero A, Esposito S. Drugs for Influenza Treatment: Is There Significant News? [J]. Frontiers in Medicine, 2019, 6. | Wrong research type |
| [332] | Qiu C, Cheng F, Ye X, Wu Z, Ning H, Liu S, et al. Study on the clinical efficacy and safety of baloxavir marboxil tablets in the treatment of influenza A [J]. Frontiers in Medicine, 2024, 11. | Wrong comparator |
| [333] | Scherger S J, Kalil A C. In persons exposed to influenza, zanamivir, oseltamivir, laninamivir, and baloxavir reduce symptomatic seasonal influenza [J]. Annals of internal medicine, 2024. | Wrong study type |
| [334] | Takazono T, Ito G, Hosogaya N, Iwanaga N, Komeda T, Kobayashi M, et al. Comparison of the Effectiveness of Baloxavir and Oseltamivir in Outpatients With Influenza B [J]. Influenza and other respiratory viruses, 2024, 18(9): e70002. | Wrong study design |
| [335] | Wei W, Huang L, Bai Y, Chang E, Liu J. The real-world safety of oseltamivir and baloxavir marboxil in children: a disproportionality analysis of the FDA adverse event reporting system [J]. Frontiers in pharmacology, 2024, 15. | Wrong study type |
| [336] | Yokoyama T, Sakaguchi H, Ishibashi T, Shishido T, Piedra P A, Sato C, et al. Baloxavir Marboxil 2% Granules in Japanese Children With Influenza An Open-label Phase 3 Study [J]. PEDIATRIC INFECTIOUS DISEASE JOURNAL, 2020, 39(8): 706-712. | Wrong comparator |
| [337] | Ohkusa Y, Sugawara T, Taniguchi K, Miyazaki C, Momoi M Y, Okabe N. Comment on: Risk of neuropsychiatric adverse events associated with the use of oseltamivir: a nationwide population-based case-crossover study [J]. Journal of Antimicrobial Chemotherapy, 2019, 74(6): 1762-1764. | Wrong intervention |
| [338] | O'Hanlon R, Shaw M L. Baloxavir marboxil: the new influenza drug on the market [J]. Current opinion in virology, 2019, 35: 14-18. | Wrong research type |
| [339] | Ntem-Mensah A D, Heil E, Pajoumand M, Rabinowitz R, Galvagno S, Schrank G. Baloxavir marboxil in combination with oseltamivir in two critically ill patients with influenza A (H1N1; 2009 strain) on veno-venous extra-corporal membranous oxygenation [J]. Open Forum Infectious Diseases, 2019, 6: S772. | Wrong research type |
| [340] | Noorsaeed S M Z. Potential Universal Influenza a Viruses Diagnostic Capture Assay [M]. 2019. | Irrelevant study |
| [341] | Ng K E. Xofluza (Baloxavir marboxil) for the treatment of acute uncomplicated influenza [J]. P and T, 2019, 44(1): 9-11. | Wrong research type |
| [342] | Nakagawa N, Lai L. A COST-EFFECTIVENESS ANALYSIS FOR INFLUENZA VIRUS INFECTION WITH OSELTAMIVIR OR BALOXAVIR IN JAPAN [J]. VALUE IN HEALTH, 2019, 22: S202-S202. | Wrong outcome |
| [343] | Nakagawa N, Lai L. PIN48 A COST-EFFECTIVENESS ANALYSIS FOR INFLUENZA VIRUS INFECTION WITH OSELTAMIVIR OR BALOXAVIR IN JAPAN [J]. Value in Health, 2019, 22: S202. | Wrong outcome |
| [344] | Nachiappan A C, Yang W T. Comparing length of stay and clinical outcomes for hospitalized patients at bridgeport hospital who received baloxavir marboxil (BM) or oseltamivir phosphate (OP) during the 2018-2019 influenza season [J]. Open Forum Infectious Diseases, 2019, 6: S326. | Wrong outcome |
| [345] | Miskulin D C. Influenza in dialysis patients: An opportunity to decrease mortality? [J]. Current opinion in nephrology and hypertension, 2019, 28(6): 607-614. | Irrelevant study |
| [346] | Mifsud E J, Hayden F G, Hurt A C. Antivirals targeting the polymerase complex of influenza viruses [J]. Antiviral research, 2019, 169: 104545. | Irrelevant study |
| [347] | McGowan D C, Balemans W, Embrechts W, Motte M, Keown J R, Buyck C, et al. Design, Synthesis, and Biological Evaluation of Novel Indoles Targeting the Influenza PB2 Cap Binding Region [J]. Journal of medicinal chemistry, 2019, 62(21): 9680-9690. | Irrelevant study |
| [348] | Locke S C, Splawn L M, Cho J C. Baloxavir marboxil: a novel cap-dependent endonuclease (CEN) inhibitor for the treatment of acute uncomplicated influenza [J]. Drugs of today (Barcelona, Spain : 1998), 2019, 55(6): 359-366. | Wrong research type |
| [349] | Lee N, Ison M G. Inhibiting viral polymerase and neuraminidase in treating influenza [J]. Journal of Infectious Diseases, 2019, 219(7): 1013-1015. | Irrelevant study |
| [350] | Koszalka P, Tilmanis D, Roe M, Vijaykrishna D, Hurt A C. Baloxavir marboxil susceptibility of influenza viruses from the Asia-Pacific, 2012-2018 [J]. Antiviral research, 2019, 164: 91-96. | Wrong population |
| [351] | Koshimichi H, Tsuda Y, Ishibashi T, Wajima T. Population Pharmacokinetic and Exposure-Response Analyses of Baloxavir Marboxil in Adults and Adolescents Including Patients With Influenza [J]. Journal of pharmaceutical sciences, 2019, 108(5): 1896-1904. | Wrong population |
| [352] | Kiso M, Yamayoshi S, Furusawa Y, Imai M, Kawaoka Y. Treatment of Highly Pathogenic H7N9 Virus-Infected Mice with Baloxavir Marboxil [J]. Viruses, 2019, 11(11). | Wrong study design |
| [353] | Kiseleva I. Will we ever be able to defeat human influenza? [J]. Open Microbiology Journal, 2019, 13(1): 313-314. | Irrelevant study |
| [354] | Kaufman M B. Pharmaceutical approval update [J]. P and T, 2019, 44(2): 42-44. | Irrelevant study |
| [355] | Kanai N, Hashimoto T, Fukuda M, Shijyo T. Acute ischemic colitis with hematochezia related to baloxavir marboxil treatment for influenza A [J]. Journal of infection and chemotherapy : official journal of the Japan Society of Chemotherapy, 2019, 25(12): 1040-1042. | Wrong research type |
| [356] | Hurt A C. Antiviral therapy for the next influenza pandemic [J]. Tropical Medicine and Infectious Disease, 2019, 4(2). | Irrelevant study |
| [357] | Honce R, Schultz-Cherry S. Impact of obesity on influenza A virus pathogenesis, immune response, and evolution [J]. Frontiers in immunology, 2019, 10(MAY). | Irrelevant study |
| [358] | Hayden F G, Shindo N. Influenza virus polymerase inhibitors in clinical development [J]. Current opinion in infectious diseases, 2019, 32(2): 176-186. | Irrelevant study |
| [359] | Gubareva L V, Mishin V P, Patel M C, Chesnokov A, Nguyen H T, De La Cruz J, et al. Assessing baloxavir susceptibility of influenza viruses circulating in the United States during the 2016/17 and 2017/18 seasons [J]. EUROSURVEILLANCE, 2019, 24(3): 13-17. | Wrong study design |
| [360] | Gaitonde D Y, Moore F C, Morgan M K. Influenza: Diagnosis and treatment [J]. American Family Physician, 2019, 100(12): 751-758. | Irrelevant study |
| [361] | Fukao K, Noshi T, Yamamoto A, Kitano M, Ando Y, Noda T, et al. Combination treatment with the cap-dependent endonuclease inhibitor baloxavir marboxil and a neuraminidase inhibitor in a mouse model of influenza A virus infection [J]. The Journal of antimicrobial chemotherapy, 2019, 74(3): 654-662. | Wrong study design |
| [362] | Fukao K, Ando Y, Noshi T, Kitano M, Noda T, Kawai M, et al. Baloxavir marboxil, a novel cap-dependent endonuclease inhibitor potently suppresses influenza virus replication and represents therapeutic effects in both immunocompetent and immunocompromised mouse models [J]. PloS one, 2019, 14(5): e0217307. | Wrong study design |
| [363] | Fontana L, Strasfeld L. Respiratory Virus Infections of the Stem Cell Transplant Recipient and the Hematologic Malignancy Patient [J]. Infectious Disease Clinics of North America, 2019, 33(2): 523-544. | Irrelevant study |
| [364] | Chua S C J H, Tan H Q, Engelberg D, Lim L H K. Alternative experimental models for studying influenza proteins, host-virus interactions and anti-influenza drugs [J]. Pharmaceuticals, 2019, 12(4). | Irrelevant study |
| [365] | Chow E J, Doyle J D, Uyeki T M. Influenza virus-related critical illness: Prevention, diagnosis, treatment [J]. Critical Care, 2019, 23(1). | Irrelevant study |
| [366] | Checkmahomed L, Mhamdi Z, Carbonneau J, Baz M, Abed Y, Boivin G. Competition experiments for the baloxavir-resistant I38T influenza a mutant [J]. Open Forum Infectious Diseases, 2019, 6: S9. | Wrong study design |
| [367] | Beigel J H, Nam H H, Adams P L, Krafft A, Ince W L, El-Kamary S S, et al. Advances in respiratory virus therapeutics – A meeting report from the 6th isirv Antiviral Group conference [J]. Antiviral research, 2019, 167: 45-67. | Irrelevant study |
| [368] | Behzadi M A, Leyva-Grado V H. Overview of current therapeutics and novel candidates against influenza, respiratory syncytial virus, and Middle East respiratory syndrome coronavirus infections [J]. Frontiers in Microbiology, 2019, 10(JUN). | Irrelevant study |
| [369] | Aoki F Y, Allen U D, Mubareka S, Papenburg J, Grant Stiver H, Evans G A. Use of antiviral drugs for seasonal influenza: Foundation document for practitioners—update 2019 [J]. JAMMI, 2019, 4(2): 60-82. | Irrelevant study |
| [370] | Expanded table: Antiviral drugs for treatment and prophylaxis of seasonal influenza 2018-2019 [J]. The Medical letter on drugs and therapeutics, 2019, 61(1563): e11-e12. | Irrelevant study |
| [371] | Antiviral drugs for treatment and prophylaxis of seasonal influenza [J]. The Medical letter on drugs and therapeutics, 2019, 61(1563): 1-4. | Irrelevant study |
| [372] | Uyeki T M. A step forward in the treatment of influenza [J]. New England Journal of Medicine, 2018, 379(10): 975-977. | Irrelevant study |
| [373] | Takashita E, Morita H, Ogawa R, Nakamura K, Fujisaki S, Shirakura M, et al. Susceptibility of influenza viruses to the novel cap-dependent endonuclease inhibitor baloxavir marboxil [J]. Frontiers in Microbiology, 2018, 9(DEC). | Wrong study design |
| [374] | Steurer J. Baloxavir marboxil in influenza patients is more effective than placebo, but not more effective than oseltamivir [J]. Praxis, 2018, 107(25): 1419-1420. | Wrong research type |
| [375] | Omoto S, Speranzini V, Hashimoto T, Noshi T, Yamaguchi H, Kawai M, et al. Characterization of influenza virus variants induced by treatment with the endonuclease inhibitor baloxavir marboxil [J]. Scientific reports, 2018, 8. | Wrong study design |
| [376] | Noshi T, Kitano M, Taniguchi K, Yamamoto A, Omoto S, Baba K, et al. In vitro characterization of baloxavir acid, a first-in-class cap-dependent endonuclease inhibitor of the influenza virus polymerase PA subunit [J]. Antiviral research, 2018, 160: 109-117. | Wrong study design |
| [377] | McKimm-Breschkin J L, Jiang S, Hui D S, Beigel J H, Govorkova E A, Lee N. Prevention and treatment of respiratory viral infections: Presentations on antivirals, traditional therapies and host-directed interventions at the 5th ISIRV Antiviral Group conference [J]. Antiviral research, 2018, 149: 118-142. | Wrong study design |
| [378] | Koshimichi H, Ishibashi T, Kawaguchi N, Sato C, Kawasaki A, Wajima T. Safety, Tolerability, and Pharmacokinetics of the Novel Anti-influenza Agent Baloxavir Marboxil in Healthy Adults: Phase I Study Findings [J]. CLINICAL DRUG INVESTIGATION, 2018, 38(12): 1189-1196. | Wrong study design |
| [379] | Kitano M, Matsuzaki T, Oka R, Baba K, Noda T, Yoshida Y, et al. Therapeutic effects of baloxavir marboxil against influenza a virus infection in ferrets [J]. Open Forum Infectious Diseases, 2018, 5: S413. | Wrong study design |
| [380] | Kawaguchi N, Koshimichi H, Ishibashi T, Wajima T. Evaluation of Drug–Drug Interaction Potential between Baloxavir Marboxil and Oseltamivir in Healthy Subjects [J]. Clinical Drug Investigation, 2018, 38(11): 1053-1060. | Wrong study design |
| [381] | Kawaguchi K, Portsmouth S, Shishido T, Uehara T, Hayden F. Exploring clinical and antiviral efficacy of baloxavir marboxil in a phase 3, randomized, double-blind, placebo-and active-controlled study of otherwise healthy adults/adolescents in seasonal influenza: Impact on regional participants, treatment time and influenza type b virus infection (capstone-1 study) [J]. Open Forum Infectious Diseases, 2018, 5: S48. | Wrong study design |
| [382] | Ison M G, Portsmouth S, Yoshida Y, Shishido T, Hayden F, Uehara T. LB16. Phase 3 Trial of Baloxavir Marboxil in High-Risk Influenza Patients (CAPSTONE-2 Study) [J]. Open Forum Infectious Diseases, 2018, 5(suppl_1): S764-S765. | Wrong study design |
| [383] | Hijano D R, Maron G, Hayden R T. Respiratory viral infections in patients with cancer or undergoing hematopoietic cell transplant [J]. Frontiers in Microbiology, 2018, 9(DEC). | Irrelevant study |
| [384] | Huang G, Tian Y, Cui W, Zhang X, Zhao Y, Liu X. Rapid health technology assessment of the novel endonuclease inhibitor baloxavir for the treatment of influenza [J]. Journal of Chemotherapy, 2024, 36(4): 267-282. | Wrong research type |
| [385] | Hayden F G, Granwehr B P. In acute uncomplicated influenza, single-dose baloxavir decreased time to symptom relief compared with placebo [J]. Annals of internal medicine, 2018, 169(12): JC63. | Wrong comparator |
| [386] | Han J, Perez J, Schafer A, Cheng H, Peet N, Rong L, et al. Influenza virus: Small molecule therapeutics and mechanisms of antiviral resistance [J]. Current medicinal chemistry, 2018, 25(38): 5115-5127. | Irrelevant study |
| [387] | Euctr E S. A Study to Assess the Safety, Pharmacokinetics, and Efficacy of Baloxavir Marboxil in Otherwise Healthy Pediatric Patients 1 to <12 Years of Age with Influenza-Like Symptoms [J]. https://trialsearchwhoint/Trial2aspx?TrialID=EUCTR2018-002169-21-ES, 2018. | Wrong comparator |
| [388] | Cole P. Baloxavir marboxil. Influenza virus cap-dependent endonuclease (CEN) inhibitor, Anti-influenza agent [J]. DRUGS OF THE FUTURE, 2018, 43(6): 379-387. | Wrong research type |
| [389] | Cole P. Baloxavir marboxil [J]. Drugs of the Future, 2018, 43(6): 379-387. | Wrong research type |
| [390] | Baloxavir marboxil (Xofluza) for treatment of influenza [J]. The Medical letter on drugs and therapeutics, 2018, 60(1561): 193-196. | Wrong research type |
| [391] | Portsmouth S, Kawaguchi K, Arai M, Tsuchiya K, Uehara T. Cap-dependent endonuclease inhibitor s-033188 for the treatment of influenza: Results from a phase 3, randomized, double-blind, placebo-and active-controlled study in otherwise healthy adolescents and adults with seasonal influenza [J]. Open Forum Infectious Diseases, 2017, 4: S734. | Wrong population |
| [392] | Koszalka P, Tilmanis D, Hurt A C. Influenza antivirals currently in late-phase clinical trial [J]. Influenza and other respiratory viruses, 2017, 11(3): 240-246. | Irrelevant study |
| [393] | Kitano M, Yamamoto A, Noshi T, Kawai M, Yoshida R, Sato A, et al. Synergistic antiviral activity of s-033188/s-033447, a novel inhibitor of influenza virus cap-dependent endonuclease, in combination with neuraminidase inhibitors in vitro [J]. Open Forum Infectious Diseases, 2017, 4: S371. | Wrong study design |
| [394] | Hussain M, Galvin H D, Haw T Y, Nutsford A N, Husain M. Drug resistance in influenza a virus: The epidemiology and management [J]. Infection and drug resistance, 2017, 10: 121-134. | Irrelevant study |
| [395] | Fukao K, Ando Y, Noshi T, Kawai M, Yoshida R, Shishido T, et al. Delayed dosing of S-033188, a novel inhibitor of influenza virus cap-dependent endonuclease, exhibited significant reduction of viral titer and mortality in mice infected with influenza a virus [J]. Open Forum Infectious Diseases, 2017, 4: S473. | Wrong study design |
| [396] | Shionogi I. A Study of S-033188 (Baloxavir Marboxil) Compared With Placebo or Oseltamivir in Otherwise Healthy Patients With Influenza [J]. clinicaltrialsgov, 2016. | Wrong study design |
| [397] | Nct. A Study of S-033188 (Baloxavir Marboxil) Compared With Placebo or Oseltamivir in Otherwise Healthy Patients With Influenza [J]. https://clinicaltrialsgov/show/NCT02954354, 2016. | Wrong study design |
| [398] | Blair W, Cox C. Current Landscape of Antiviral Drug Discovery [J]. F1000Research, 2016, 5. | Irrelevant study |
| [399] | Zhang M, Chen X. Application of (Z)-N-(3-((5-chloro-4-(1H-indol-3-yl)pyrimidin-2-yl)amino)phenyl)-3-((Z)-((E)-4-(dimethylamino)-1-hydroxybut-2-en-1-ylidene)amino)benzimidic acid (THZ2) for preparing anti-influenza virus medicine and avoiding or reducing infection rate of influenza virus, CN117942343-A [P/OL]. 2024-06-01]. | Irrelevant study |
| [400] | Zhang M, Chen X. Use of CVT-313 in preparation of anti-influenza virus drugs and e.g. for avoiding or reducing infection rate of influenza virus, and inhibiting influenza virus growth, influenza virus proliferation, synthesis of influenza virus RNA and influenza virus nucleoprotein synthesis, CN117959304-A [P/OL]. 2024-06-01]. | Irrelevant study |
| [401] | Yu Y, Chen X, Chen S. Use of ruxolitinib for treating influenza virus infection, reducing infection rate, inhibiting growth and replication of influenza virus and improving survival rate of body infected by influenza virus including influenza A, influenza B virus and avian influenza virus, CN117838696-A [P/OL]. 2024-05-23]. | Irrelevant study |
| [402] | Yu Y, Chen X. Use of oclacitinib in preparing drug for treating influenza virus infection, avoiding or reducing infection rate of influenza virus, and inhibiting growth of influenza viruses, influenza virus replication, and inflammatory effects produced by influenza viruses, CN117838698-A [P/OL]. 2024-05-11]. | Irrelevant study |
| [403] | Yang X, Lu L, Jiang S, Fu Y, Li P, Xu W. Inhibiting influenza virus, by administering anhydride-treated protein and optionally drug including oseltamivir, zanamivir, peramivir and lamivtide to influenza virus, and mixing acid anhydride solution with protein solution, CN109675042-A CN109675042-B [P/OL]. 2019-08-10]. | Wrong intervention |
| [404] | Xu A, Zhou N, Zhang X. New 5-fluoro-1H-pyrazolo(3,4-b)pyridine derivative used in pharmaceutical composition and used in combination with baloxavir and oseltamivir for preparing medicament related to treatment of influenza virus infection in mammal, CN112724130-A [P/OL]. 2021-06-20]. | Wrong study design |
| [405] | Shi N, Zheng Z, Xia Q. New replication-deficient medicine-resistant influenza virus i.e. influenza virus rescued by reverse genetics operation useful in e.g. preparing medicine for preventing and treating influenza virus infection comprises medicine resistance mutation and premature termination codons mutation, CN113755456-A | Irrelevant study |
| [406] | Planz O. Method of treating human subject having viral infection caused by influenza A or influenza B virus comprises administering subject with MEK inhibitor CI-1040 or its salt or derivative, US2019298669-A1 | Irrelevant study |
| [407] | Ludwig S, Planz O, Ludewig S. Use of p90 ribosomal S6 kinase inhibitor for prophylaxing or treating viral disease i.e. infection caused by negative strand RNA virus, preferably influenza virus, WO2020188034-A1 | Irrelevant study |
| [408] | Lian X, Chen X, Zhang M. Use of 3-[[6-(2-methoxyphenyl)-4-pyrimidinyl]amino]benzenemethanesulfonamide in preparing medicine for resisting influenza virus including influenza A virus hemagglutinin (H)1, neuraminidase (N)1 (H1N1) subtype, H2N2 subtype, H3N2 subtype and avian influenza virus, CN117919247-A [P/OL]. 2024-06-01]. | Irrelevant study |
| [409] | Chen X, Chen S, Yu Y. Use of peficitinib in preparing medicine for treating influenza virus infection, where influenza virus comprises influenza A and/or influenza B virus, preferably influenza A virus H1N1 subtype, H2N2 subtype, H3N2 subtype and avian influenza virus of all subtypes, CN117838689-A [P/OL]. 2024-05-11]. | Irrelevant study |
| [410] | Hanaki N, Sakaniwa R, Moromizato T, Miyata J, Ishimura K, Noguchi M, et al. Efficacy of Pharmacotherapy for Seasonal Influenza in Young and Middle-aged Adults: A Systematic Review and Network Meta-analysis [J]. Internal Medicine, 2024, 63(21): 2913-2922. | Wrong study type |
| [411] | Bridgeman M B, Mansukhani R P. OTC case studies: Respiratory health [Z]. 2019 | Wrong study type |
| [412] | Ahmed A R. H1N1 with fatal viral septicemia in a normal child: A case report [J]. Respiratory Medicine Case Reports, 2020, 30. | Wrong study type |
| [413] | Harada N, Shibata W, Koh H, Takashita E, Fujisaki S, Okamura H, et al. Successful treatment with baloxavir marboxil of a patient with peramivir-resistant influenza A/H3N2 with a dual E119D/R292K substitution after allogeneic hematopoietic cell transplantation: A case report [J]. BMC Infectious Diseases, 2020, 20(1). | Wrong study type |
| [414] | Huang B R, Lin Y L, Wan C K, Wu J T, Hsu C Y, Chiu M H, et al. Co-infection of influenza B virus and SARS-CoV-2: A case report from Taiwan [J]. Journal of Microbiology, Immunology and Infection, 2021, 54(2): 336-338. | Wrong study type |
| [415] | Kolosova N P, Ilyicheva T N, Svyatchenko S V, Danilenko A V, Onkhonova G S, Ivanova K I, et al. INITIAL AND SEVERE CASES OF INFLUENZA IN 2020-2022 AND POPULATION IMMUNITY PRIOR TO EPIDEMIC SEASON [J]. Medical Immunology (Russia), 2022, 24(6): 1219-1226. | Wrong study type |
| [416] | Puzelli S, Facchini M, Piacentini S, Di Mario G, Colucci M E, Calzoletti L, et al. Characterization of an influenza B virus isolated from a fatal case of myocarditis in a pediatric patient in Italy [J]. Journal of Infection and Public Health, 2024, 17(3): 417-420. | Wrong study type |
| [417] | Harada N, Shibata W, Koh H, Takashita E, Fujisaki S, Okamura H, et al. Successful treatment with baloxavir marboxil of a patient with peramivir-resistant influenza A/H3N2 with a dual E119D/R292K substitution after allogeneic hematopoietic cell transplantation: a case report [J]. BMC INFECTIOUS DISEASES, 2020, 20(1). | Wrong study type |
| [418] | Younossi Z M, Koenig A B, Abdelatif D, Fazel Y, Henry L, Wymer M. Global epidemiology of nonalcoholic fatty liver disease-Meta-analytic assessment of prevalence, incidence, and outcomes [J]. Hepatology, 2016, 64(1): 73-84. | Wrong study type |
| [419] | Taieb V, Borkowska K, Ma F, Tone K, Ikeoka H. A network meta-analysis of the efficacy and safety of baloxavir marboxil suggests a better control of the viral load versus neuraminidase inhibitors in the treatment of influenza in otherwise healthy patients [J]. Value in Health, 2018, 21: S148. | Wrong study type |
| [420] | Taieb V, Borkowska K, Jablonska K, Hill M, Ikeoka H. PIN7 A NETWORK META-ANALYSIS OF THE EFFICACY AND SAFETY OF BALOXAVIR MARBOXIL VERSUS NEURAMINIDASE INHIBITORS IN THE TREATMENT OF INFLUENZA VIRUS INFECTION IN HIGH-RISK PATIENTS [J]. Value in Health, 2019, 22: S196. | Wrong study type |
| [421] | Taieb V, Ikeoka H, Ma F F, Borkowska K, Aballéa S, Tone K, et al. A network meta-analysis of the efficacy and safety of baloxavir marboxil versus neuraminidase inhibitors for the treatment of influenza in otherwise healthy patients [J]. Current Medical Research and Opinion, 2019, 35(8): 1355-1364. | Wrong study type |
| [422] | Taieb V, Wojciechowski P, Jabłońska K, Aballea S, Hill M, Ikeoka H. PIN4 A NETWORK META-ANALYSIS OF THE EFFICACY AND SAFETY OF BALOXAVIR MARBOXIL VERSUS NEURAMINIDASE INHIBITORS IN THE TREATMENT OF INFLUENZA VIRUS INFECTION IN OTHERWISE HEALTHY AND HIGH-RISK ADULTS [J]. Value in Health, 2019, 22: S640. | Wrong study type |
| [423] | Taieb V, Ikeoka H, Ma F, Borkowska K, Aballea S, Tone K, et al. A network meta-analysis of the efficacy and safety of baloxavir marboxil versus neuraminidase inhibitors for the treatment of influenza in otherwise healthy patients [J]. Terapevticheskii arkhiv, 2020, 92(11): 122-131. | Wrong study type |
| [424] | Kuo Y C, Lai C C, Wang Y H, Chen C H, Wang C Y. Clinical efficacy and safety of baloxavir marboxil in the treatment of influenza: A systematic review and meta-analysis of randomized controlled trials [J]. Journal of Microbiology, Immunology and Infection, 2021, 54(5): 865-875. | Wrong study type |
| [425] | Liu J W, Lin S H, Wang L C, Chiu H Y, Lee J A. Comparison of Antiviral Agents for Seasonal Influenza Outcomes in Healthy Adults and Children: A Systematic Review and Network Meta-analysis [J]. JAMA Network Open, 2021, 4(8) | Wrong study type |
| [426] | Taieb V, Ikeoka H, Wojciechowski P, Jablonska K, Aballea S, Hill M, et al. Efficacy and safety of baloxavir marboxil versus neuraminidase inhibitors in the treatment of influenza virus infection in high-risk and uncomplicated patients–a Bayesian network meta-analysis [J]. Current Medical Research and Opinion, 2021, 37(2): 225-244. | Wrong study type |
| [427] | Okoli G N, Lam O L T, Reddy V K, Al-Yousif Y, Racovitan F, Askin N. An overview of the characteristics and methodological standards across systematic reviews with Meta-analysis of efficacy/effectiveness of influenza antiviral drugs [J]. Current Medical Research and Opinion, 2022, 38(12): 2035-2046. | Wrong study type |
| [428] | Zhao Y, Huang G, He W, Sun Q, Zhao X, Li D, et al. Efficacy and safety of single-dose antiviral drugs for influenza treatment: A systematic review and network meta-analysis [J]. JOURNAL OF MEDICAL VIROLOGY, 2022, 94(7): 3270-3302. | Wrong study type |
| [429] | Shiraishi C, Kato H, Hagihara M, Asai N, Iwamoto T, Mikamo H. Comparison of clinical efficacy and safety of baloxavir marboxil versus oseltamivir as the treatment for influenza virus infections: A systematic review and meta-analysis [J]. Journal of infection and chemotherapy : official journal of the Japan Society of Chemotherapy, 2023. | Wrong study type |
| [430] | Okoli G N, Lam O L T, Reddy V K, Al-Yousif Y, Racovitan F, Askin N. An overview of the characteristics and methodological standards across systematic reviews with Meta-analysis of efficacy/effectiveness of influenza antiviral drugs [J]. Current Medical Research and Opinion, 2022, 38(12): 2035-2046. | Wrong study type |
| [431] | Zhao Y, Huang G, He W, Sun Q, Zhao X, Li D, et al. Efficacy and safety of single-dose antiviral drugs for influenza treatment: A systematic review and network meta-analysis [J]. Journal of Medical Virology, 2022, 94(7): 3270-3302. | Wrong study type |
| [432] | Shiraishi C, Kato H, Hagihara M, Asai N, Iwamoto T, Mikamo H. Comparison of clinical efficacy and safety of baloxavir marboxil versus oseltamivir as the treatment for influenza virus infections: A systematic review and meta-analysis [J]. Journal of infection and chemotherapy : official journal of the Japan Society of Chemotherapy, 2023. | Wrong study type |
| [433] | Huang Guangliang. Evaluation of the Effectiveness, Safety and Economy of Antiviral Drugs in the Treatment of Influenza [D], 2023. | Wrong study type |
| [434] | Aldhaeefi M, Rungkitwattanakul D, Saltani I, Muirhead A, Ruehman A J, Hawkins W A, et al. Update and narrative review of avian influenza (H5N1) infection in adult patients [J]. Pharmacotherapy, 2024, 44(11): 870-879. | Wrong study type |
| [435] | Chakraborty S, Chauhan A. Fighting the flu: a brief review on anti-influenza agents [J]. Biotechnol Genet Eng Rev, 2024, 40(2): 858-909. | Wrong study type |
| [436] | Gao Y, Zhao Y, Liu M, Luo S, Chen Y, Chen X, et al. Antivirals for treatment of non-severe influenza: a systematic review and network meta-analysis of randomized controlled trials [Z]. 2024.10.1101/2024.05.28.24307936 | Wrong study type |
| [437] | Hanaki N, Sakaniwa R, Moromizato T, Miyata J, Ishimura K, Noguchi M, et al. Efficacy of Pharmacotherapy for Seasonal Influenza in Young and Middle-aged Adults: A Systematic Review and Network Meta-analysis [J]. INTERNAL MEDICINE, 2024, 63(21): 2913-2922. | Wrong study type |
| [438] | He J, Kam Y W. Insights from Avian Influenza: A Review of Its Multifaceted Nature and Future Pandemic Preparedness [J]. Viruses, 2024, 16(3). | Wrong study type |
| [439] | Shiraishi C, Kato H, Hagihara M, Asai N, Iwamoto T, Mikamo H. Comparison of clinical efficacy and safety of baloxavir marboxil versus oseltamivir as the treatment for influenza virus infections: A systematic review and meta-analysis [J]. J Infect Chemother, 2024, 30(3): 242-249. | Wrong study type |
| [440] | Zhao Y, Gao Y, Guyatt G, Uyeki T M, Liu P, Liu M, et al. Antivirals for post-exposure prophylaxis of influenza: a systematic review and network meta-analysis [J]. Lancet, 2024, 404(10454): 764-772. | Wrong study type |
| [441] | Yoshii N, Tochino Y, Fujioka M, Sakazaki H, Maruyama N, Asai K, et al. The comparison of the efficacy of baloxavir and neuraminidase inhibitors for patients with influenza a in clinical practice [J]. Internal Medicine, 2020, 59(12): 1509-1513. | Wrong comparator |
| [442] | Chong Y, Kawai N, Tani N, Bando T, Takasaki Y, Shindo S, et al. Virological and clinical outcomes in outpatients treated with baloxavir or oseltamivir: A Japanese multicenter study in the 2019–2020 influenza season [J]. Antiviral research, 2021, 192. | Wrong population |
| [443] | Ishiguro N, Morioka I, Nakano T, Furukawa M, Tanaka S, Kinoshita M, et al. Clinical and virological outcomes with baloxavir compared with oseltamivir in pediatric patients aged 6 to &lt; 12 years with influenza: an open-label, randomized, active-controlled trial protocol [J]. BMC INFECTIOUS DISEASES, 2021, 21(1). | Full text not  obtainable |
| [444] | Cagas S, Gupta S, Han J, McIntosh M, Collins C, Sun C, et al. Efficacy and Safety of Baloxavir Marboxil for the Treatment of Influenza Virus Infection in Patients with Chronic Lung Disease: A Subgroup Analysis of CAPSTONE-2 [J]. American journal of respiratory and critical care medicine, 2022, 205(1). | Wrong population |
| [445] | Kumar D, Ison M G, Mira J-P, Welte T, Ha J H, Hui D S, et al. Combining baloxavir marboxil with standard-of-care neuraminidase inhibitor in patients hospitalised with severe influenza (FLAGSTONE): a randomised, parallel-group, double-blind, placebo-controlled, superiority trial [J]. LANCET INFECTIOUS DISEASES, 2022, 22(5): 718-730. | Wrong comparator |
| [446] | Shinno K, Takeuchi M, Kawakami K. Association between Baloxavir Marboxil Prescription for Children with Influenza B Infections and Short-Term Healthcare Consumption in Japan during the 2018-2019 Influenza Season [J]. Journal of the Pediatric Infectious Diseases Society, 2022, 11(7): 310-315. | Wrong outcome |
| [447] | Barnes A, Ringer M, Malinis M, McManus D, Davis M W, Shah S, et al. Clinical Outcomes of Baloxavir vs. Oseltamivir in Transplant and Other Immunocompromised Patients [J]. American Journal of Transplantation, 2023, 23(6): S1110. | Wrong population |
| [448] | Hirotsu N, Sakaguchi H, Fukao K, Kojima S, Piedra P A, Tsuchiya K, et al. Baloxavir safety and clinical and virologic outcomes in influenza virus-infected pediatric patients by age group: age-based pooled analysis of two pediatric studies conducted in Japan [J]. BMC PEDIATRICS, 2023, 23(1). | Wrong comparator |
| [449] | Li J, Wagatsuma K, Sun Y, Sato I, Kawashima T, Saito T, et al. Factors associated with viral RNA shedding and evaluation of potential viral infectivity at returning to school in influenza outpatients after treatment with baloxavir marboxil and neuraminidase inhibitors during 2013/2014–2019/2020 seasons in Japan: an observational study [J]. BMC Infectious Diseases, 2023, 23(1). | Wrong outcome |
| [450] | Liao G, Xia M, Jiang Y, Chen H, Liao W, Peng J, et al. Prospective observational study of baloxavir marboxil in adults and adolescents with uncomplicated influenza from China [J]. Frontiers in Microbiology, 2023, 14. | Wrong population |
| [451] | Best J H, Sadeghi M, Sun X, Seetasith A, Albensi L, Joshi S, et al. Household Influenza Transmission and Healthcare Resource Utilization Among Patients Treated with Baloxavir vs Oseltamivir: A United States Outpatient Prospective Survey [J]. Infectious diseases and therapy, 2024. | Wrong outcome |
| [452] | Cai J, Wang H, Ye X, Lu S, Tan Z, Li Z, et al. Real-world effectiveness and safety of Baloxavir Marboxil or Oseltamivir in outpatients with uncomplicated influenza A: an ambispective, observational, multi-center study [J]. Frontiers in Microbiology, 2024, 15. | Wrong population |
| [453] | Goto T, Kawai N, Bando T, Takasaki Y, Shindo S, Tani N, et al. Virological and clinical outcomes in outpatients treated with baloxavir or neuraminidase inhibitors for A(H3N2) influenza: A multicenter study of the 2022–2023 season [J]. Antiviral research, 2024, 224. | Wrong population |
| [454] | Ikematsu H, Baba T, Saito M M, Kinoshita M, Miyazawa S, Hata A, et al. Comparative Effectiveness of Baloxavir Marboxil and Oseltamivir Treatment in Reducing Household Transmission of Influenza: A Post Hoc Analysis of the BLOCKSTONE Trial [J]. Influenza and other respiratory viruses, 2024, 18(5): e13302. | Wrong outcome |
| [455] | Efficacy of Baloxavir Against Influenza in Hospitalized Patients: the INFLUENT Study (INpatients InFLUENza Treatment) [J]. https://clinicaltrialsgov/ct2/show/NCT06653569, 2024. | Wrong study type |
| [456] | Hayden F G, Sugaya N, Hirotsu N, Lee N, de Jong M D, Hurt A C, et al. Baloxavir Marboxil for Uncomplicated Influenza in Adults and Adolescents [J]. New England journal of medicine, 2018, 379(10): 913‐923. | Be included in the study |
| [457] | Baker J, Block S L, Matharu B, Burleigh Macutkiewicz L, Wildum S, Dimonaco S, et al. Baloxavir marboxil single-dose treatment in influenza-infected children: A randomized, double-blind, active controlled phase 3 safety and efficacy trial (miniSTONE-2) [J]. Pediatric Infectious Disease Journal, 2020, 39(8): 700-705. | Be included in the study |
| [458] | Ison M G, Portsmouth S, Yoshida Y, Shishido T, Mitchener M, Tsuchiya K, et al. Early treatment with baloxavir marboxil in high-risk adolescent and adult outpatients with uncomplicated influenza (CAPSTONE-2): a randomised, placebo-controlled, phase 3 trial [J]. The Lancet Infectious diseases, 2020, 20(10): 1204-1214. | Be included in the study |
| [459] | Kakuya F, Okubo H, Fujiyasu H, Kurisawa M J, Kinebuchi T. Clinical effectiveness of baloxavir marboxil against influenza in three seasons [J]. Pediatrics international : official journal of the Japan Pediatric Society, 2022, 64(1): e15169. | Be included in the study |
| [460] | Saito R, Osada H, Wagatsuma K, Chon I, Sato I, Kawashima T, et al. Duration of fever and symptoms in children after treatment with baloxavir marboxil and oseltamivir during the 2018-2019 season and detection of variant influenza a viruses with polymerase acidic subunit substitutions [J]. Antiviral research, 2020, 183: 104951. | Be included in the study |
| [461] | Sato M, Takashita E, Katayose M, Nemoto K, Sakai N, Hashimoto K, et al. Detection of Variants With Reduced Baloxavir Marboxil Susceptibility After Treatment of Children With Influenza A During the 2018-2019 Influenza Season [J]. The Journal of infectious diseases, 2020, 222(1): 121-125. | Be included in the study |
| [462] | Wagatsuma K, Saito R, Chon I, Phyu W W, Fujio K, Kawashima T, et al. Duration of fever and symptoms in influenza-infected children treated with baloxavir marboxil during the 2019-2020 season in Japan and detection of influenza virus with the PA E23K substitution [J]. Antiviral research, 2022, 201: 105310. | Be included in the study |
| [463] | Ge X, Chen Y, Wu W, Lu J, Wang Y, Li Z. Safety and effectiveness of baloxavir marboxil and oseltamivir for influenza in children: a real-world retrospective study in China [J]. Frontiers in Pediatrics, 2024, 12. | Be included in the study |
