# Supplementary material for: Clinical efficacy and Safety of Baloxavir Marboxil compared with Oseltamivir against influenza virus in children: A systematic review and meta-analysis
Source: PLoS One. 2025 Jun 23;20(6):e0326777. doi: 10.1371/journal.pone.0326777 (PMC12185026; doi:10.1371/journal.pone.0326777)
Supplement: S2 Table — (DOCX) [file pone.0326777.s002.docx]

**Supplementary Material Table S2**

**The Newcastle-Ottawa Scale score of cohort studies.**

| study | Selection | | | | Comparability | Exposure | | | scores |
| --- | --- | --- | --- | --- | --- | --- | --- | --- | --- |
|  | Is the case definition adequate | Representativeness of the cases | Selection of Controls | Definition of Controls | Comparability of cases and controls on the basis of the design or analysis | Ascertainment of exposure | Same method of ascertainment for cases and controls | Non-Response rate |  |
| Saito 2020 | ☆ | ☆ | ☆ | ☆ | ☆ |  | ☆ |  | 7 |
| Wagatsuma 2020 | ☆ | ☆ |  | ☆ | ☆ |  | ☆ |  | 6 |
| Sato 2021 | ☆ | ☆ | ☆ | ☆ | ☆ |  | ☆ |  | 7 |
| FujioKakuya 2022 | ☆ | ☆ |  | ☆ | ☆ |  | ☆ |  | 6 |
| Ge, X.2024 | ☆ | ☆ | ☆ | ☆ | ☆ |  | ☆ |  | 7 |

**Quality assessment of RCT**

| study | Random sequence generation (selection bias) | Allocation concealment (selection bias) | Blinding of participants and personnel (performance bias) | Blinding of outcome assessment (detection bias) | Incomplete outcome data (attrition bias) | Selective reporting (reporting bias) | Other bias |
| --- | --- | --- | --- | --- | --- | --- | --- |
| Hayden 2018 | Low risk | Low risk | Low risk | Low risk | Low risk | Low risk | Low risk |
| Baker 2020 | Low risk | Unclear risk | Unclear risk | Unclear risk | Low risk | Low risk | Low risk |
| Ison 2020 | Low risk | Unclear risk | Low risk | Low risk | Low risk | Low risk | Low risk |

**Evaluation of the GRADE evidence level for the relief time of influenza symptoms
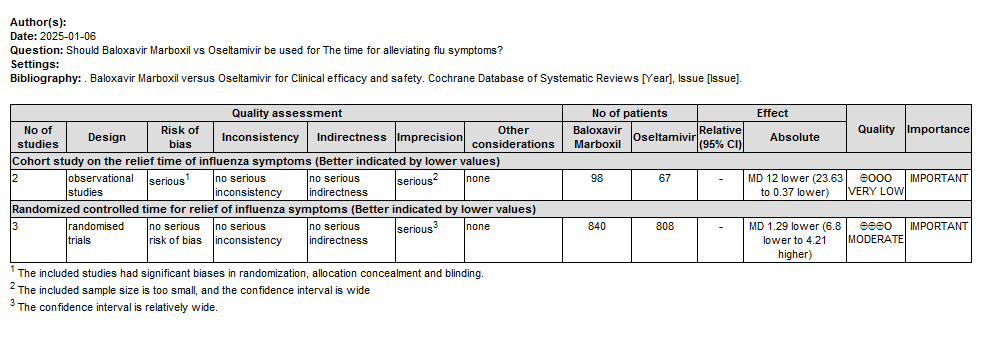
**

**Evaluation of the GRADE evidence level for the duration of fever**

**
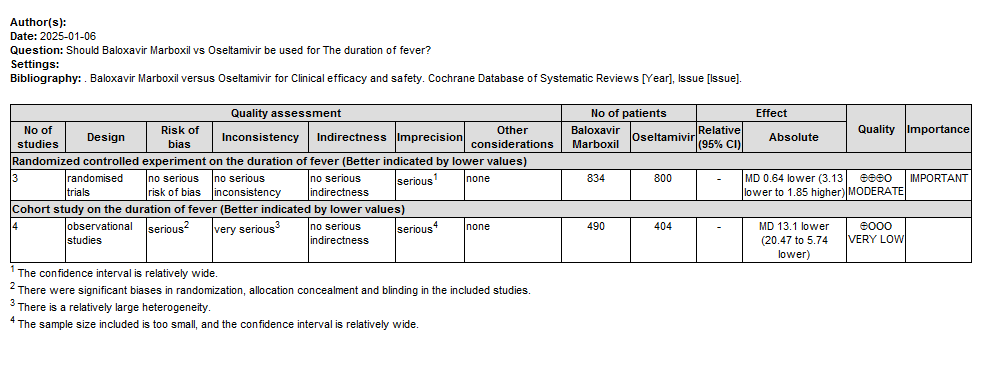
**

**Evaluation of the virus titer GRADE evidence level on the second day**

**
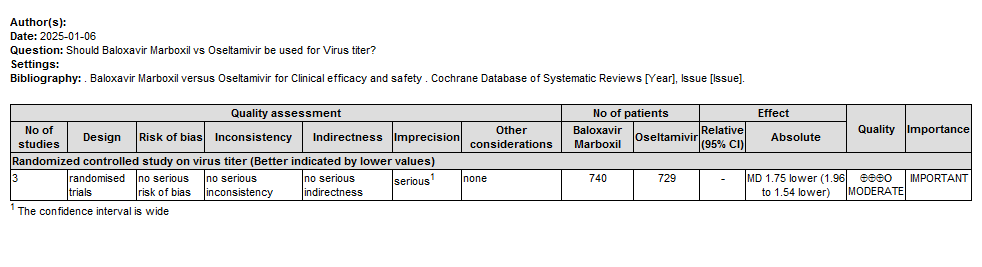
**

**Evaluation of the GRADE evidence level of the viral RNA load on the second day
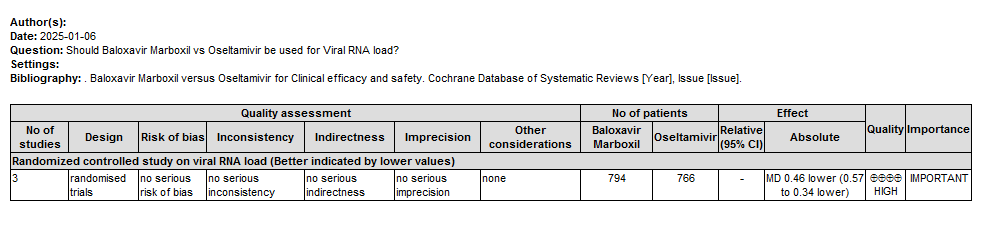
**

**Evaluation of the GRADE evidence level of adverse reactions
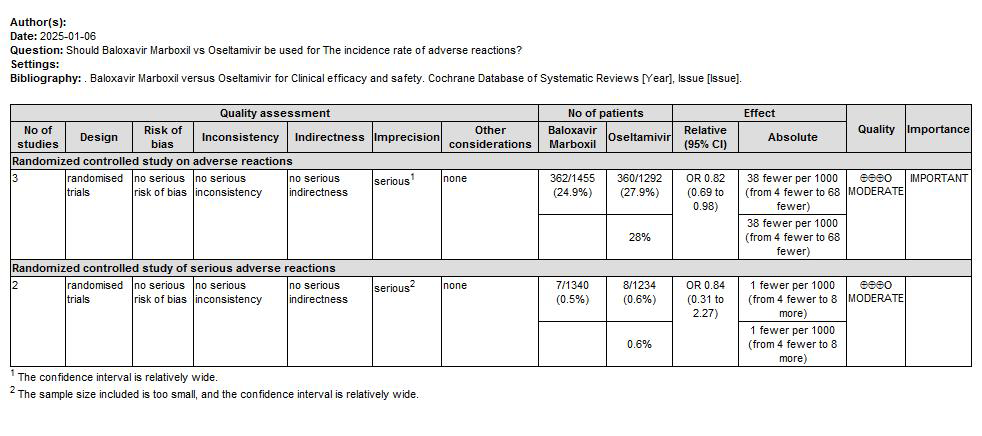
**
