# Supplementary material for: Clinical efficacy and Safety of Baloxavir Marboxil compared with Oseltamivir against influenza virus in children: A systematic review and meta-analysis
Source: PLoS One. 2025 Jun 23;20(6):e0326777. doi: 10.1371/journal.pone.0326777 (PMC12185026; doi:10.1371/journal.pone.0326777)
Supplement: S3 Table — (DOCX) [file pone.0326777.s003.docx]

**Supplementary Table S3. Analyzed data**

Time to remission of flu symptoms(RCT)

| ID | Author | year | BXM | | | OS | | |
| --- | --- | --- | --- | --- | --- | --- | --- | --- |
|  |  |  | Mean | SD | Total | Mean | SD | Total |
| 1 | Hayden | 2018 | 53.5 | 45.59 | 375 | 53.8 | 35.54 | 377 |
| 2 | Baker | 2020 | 138.1 | 96.6 | 80 | 150 | 113.7 | 43 |
| 3 | Ison | 2020 | 77 | 113 | 385 | 85.6 | 141.3 | 388 |

Time to remission of flu symptoms(cohort study)

| ID | Author | year | BXM | | | OS | | |
| --- | --- | --- | --- | --- | --- | --- | --- | --- |
|  |  |  | Mean | SD | Total | Mean | SD | Total |
| 1 | Saito | 2020 | 107.5 | 36.5 | 32 | 119.5 | 27.4 | 17 |
| 2 | Wagatsuma | 2022 | 117.6 | 38.4 | 66 | 129.2 | 43.2 | 50 |

Time to regression of fever(RCT)

| ID | Author | year | BXM | | | OS | | |
| --- | --- | --- | --- | --- | --- | --- | --- | --- |
|  |  |  | Mean | SD | Total | Mean | SD | Total |
| 1 | Hayden | 2018 | 24.4 | 21.49 | 369 | 24 | 18.69 | 374 |
| 2 | Baker | 2020 | 41.2 | 75.04 | 80 | 46.8 | 54.59 | 43 |
| 3 | Ison | 2020 | 30.8 | 25.78 | 385 | 34.3 | 42.8 | 383 |

Time to regression of fever(Cohort Study)

| ID | Author | year | BXM | | | OS | | |
| --- | --- | --- | --- | --- | --- | --- | --- | --- |
|  |  |  | Mean | SD | Total | Mean | SD | Total |
| 1 | Saito | 2020 | 25.3 | 20.7 | 34 | 28.8 | 22.6 | 17 |
| 2 | Fujio | 2022 | 22 | 12.24 | 144 | 33.5 | 32.85 | 91 |
| 3 | Wagatsuma | 2022 | 20 | 14.9 | 66 | 31.5 | 29.4 | 50 |
| 4 | Ge | 2024 | 34.56 | 15.37 | 246 | 54.96 | 16.32 | 246 |

Change in viral titer from baseline to the next day(RCT)

| ID | Author | year | BXM | | | OS | | |
| --- | --- | --- | --- | --- | --- | --- | --- | --- |
|  |  |  | Mean | SD | Total | Mean | SD | Total |
| 1 | Hayden | 2018 | -4.39 | 2.07 | 340 | -2.53 | 2.03 | 348 |
| 2 | Baker | 2020 | -3.59 | 1.34 | 64 | -1.79 | 1.54 | 37 |
| 3 | Ison | 2020 | -3.36 | 2.21 | 336 | -1.76 | 2.2 | 344 |

Change in viral titer from baseline to the next day(Cohort Study)

| ID | Author | year | BXM | | | OS | | |
| --- | --- | --- | --- | --- | --- | --- | --- | --- |
|  |  |  | Mean | SD | Total | Mean | SD | Total |
| 1 | Sato | 2021 | -4.7 | 2.15 | 20 | -2.8 | 1.56 | 16 |

Change in viral RNA load from baseline to the next day(RCT)

| ID | Author | year | BXM | | | OS | | |
| --- | --- | --- | --- | --- | --- | --- | --- | --- |
|  |  |  | Mean | SD | Total | Mean | SD | Total |
| 1 | Hayden | 2018 | -1.61 | 1.06 | 355 | -1.1 | 1.1 | 354 |
| 2 | Baker | 2020 | -1.74 | 1.13 | 70 | -1.12 | 1.12 | 39 |
| 3 | Ison | 2020 | -1.13 | 1.2 | 369 | -0.76 | 1.18 | 373 |

Change in viral RNA load from baseline to the next day(Cohort Study)

| ID | Author | year | BXM | | | OS | | |
| --- | --- | --- | --- | --- | --- | --- | --- | --- |
|  |  |  | Mean | SD | Total | Mean | SD | Total |
| 1 | Sato | 2021 | -3.5 | 0.59 | 20 | -1.9 | 1.26 | 16 |

Incidence of any adverse reactions(RCT)

| ID | Author | year | BXM | | OS | |
| --- | --- | --- | --- | --- | --- | --- |
|  |  |  | Events | Total | Events | Total |
| 1 | Hayden | 2018 | 126 | 610 | 127 | 513 |
| 2 | Baker | 2020 | 53 | 115 | 31 | 58 |
| 3 | Ison | 2020 | 183 | 730 | 202 | 721 |

Incidence of serious adverse reactions(RCT)

| ID | Author | year | BXM | | OS | |
| --- | --- | --- | --- | --- | --- | --- |
|  |  |  | Events | Total | Events | Total |
| 1 | Hayden | 2018 | 2 | 610 | 0 | 513 |
| 2 | Ison | 2020 | 5 | 730 | 8 | 721 |
